# Annual Variant-Targeted Vaccination to Prevent Severe COVID-19 in Cohorts With Vaccine-Derived and Hybrid Immunity

**DOI:** 10.1093/cid/ciaf124

**Published:** 2025-03-14

**Authors:** J Daniel Kelly, Katherine J Hoggatt, Nathan C Lo, Samuel Leonard, W John Boscardin, Hye Sun Kim, Emily N Lum, Charles C Austin, Amy L Byers, Phyllis C Tien, Peter C Austin, Dawn M Bravata, Salomeh Keyhani

**Affiliations:** Center for Data to Discovery and Delivery Innovation (3DI), San Francisco Veterans Affairs Health Care System, San Francisco, California, USA; Department of Medicine, University of California, San Francisco, California, USA; Department of Epidemiology and Biostatistics, University of California, San Francisco, California, USA; F.I. Proctor Foundation, University of California, San Francisco, California, USA; Center for Data to Discovery and Delivery Innovation (3DI), San Francisco Veterans Affairs Health Care System, San Francisco, California, USA; Department of Medicine, University of California, San Francisco, California, USA; Division of Infectious Diseases and Geographic Medicine, Department of Medicine, Stanford University, Stanford, California, USA; Center for Data to Discovery and Delivery Innovation (3DI), San Francisco Veterans Affairs Health Care System, San Francisco, California, USA; Department of Epidemiology and Biostatistics, University of California, San Francisco, California, USA; Center for Data to Discovery and Delivery Innovation (3DI), San Francisco Veterans Affairs Health Care System, San Francisco, California, USA; Center for Data to Discovery and Delivery Innovation (3DI), San Francisco Veterans Affairs Health Care System, San Francisco, California, USA; Center for Data to Discovery and Delivery Innovation (3DI), San Francisco Veterans Affairs Health Care System, San Francisco, California, USA; Department of Veterans Affairs Health Services and Development (HSR&D), Center for Health Information and Communication (CHIC), Richard L. Roudebush Veterans Affairs Medical Center, Indianapolis, Indiana, USA; Department of Medicine, Richard L. Roudebush Veterans Affairs Medical Center, Indianapolis, Indiana, USA; Center for Data to Discovery and Delivery Innovation (3DI), San Francisco Veterans Affairs Health Care System, San Francisco, California, USA; Department of Psychiatry, Weill Institute for Neurosciences, University of California, San Francisco, California, USA; Center for Data to Discovery and Delivery Innovation (3DI), San Francisco Veterans Affairs Health Care System, San Francisco, California, USA; Department of Medicine, University of California, San Francisco, California, USA; Cardiovascular Research Program, Institute for Clinical Evaluative Sciences, Toronto, Ontario, Canada; Department of Veterans Affairs Health Services and Development (HSR&D), Center for Health Information and Communication (CHIC), Richard L. Roudebush Veterans Affairs Medical Center, Indianapolis, Indiana, USA; Department of Medicine, Richard L. Roudebush Veterans Affairs Medical Center, Indianapolis, Indiana, USA; Department of Medicine, Indiana University School of Medicine, Indianapolis, Indiana, USA; William M. Tierney Center for Health Services Research, Regenstrief Institute, Indianapolis, Indiana, USA; Center for Data to Discovery and Delivery Innovation (3DI), San Francisco Veterans Affairs Health Care System, San Francisco, California, USA; Department of Medicine, University of California, San Francisco, California, USA

**Keywords:** booster-vaccinated adults, annual variant-targeted COVID-19 vaccination, hybrid immunity, severe COVID-19 illness, COVID-19 pneumonia

## Abstract

**Background:**

Current coronavirus disease 2019 (COVID-19) vaccine recommendations in the United States (US) provide guidance for adults to receive at least annual variant-targeted vaccination. We sought to estimate the strength and durability of protection from annual variant-targeted vaccination against severe COVID-19 illness in individuals with vaccine-derived and hybrid immunity.

**Methods:**

We emulated a target trial using an electronic health record–based, propensity score–matched (1:1) cohort of US Veterans. Booster-vaccinated adults were eligible for a variant-targeted messenger RNA (mRNA) booster starting 1 September 2022. Matched sets of those who did and did not receive the variant-targeted booster dose were identified on a weekly basis, and the cohort was followed until 31 August 2023. Outcomes were hospitalization due to COVID-19 pneumonia and in-hospital severe illness. We fit Cox models, overall and stratified by last documented severe acute respiratory syndrome coronavirus 2 infection (pre-Omicron, Omicron), to estimate relative vaccine effectiveness (rVE).

**Results:**

The propensity score–matched cohort consisted of 1 576 626 COVID-19 booster-vaccinated adults. Estimates of rVE from variant-targeted mRNA booster against hospitalization due to COVID-19 pneumonia were significant and similar in the cohort with vaccine-derived immunity (rVE, 29% [95% confidence interval {CI}, 25%–34%]) and cohort with hybrid immunity (rVE, 38% [95% CI, 27%–47%]). These protective gains were significant from 0 to 6 months but not 6 to 12 months after vaccination and during pre-XBB and XBB variant eras. Findings were similar for in-hospital severe illness.

**Conclusions:**

In cohorts with vaccine-derived and hybrid immunity, modest but significant gains in protection against hospitalization and severe COVID-19 illness were conferred by the annual variant-targeted booster dose but not sustained beyond 6 months.

## Teaser

Among US Veterans with hybrid immunity for COVID-19, annual variant-targeted booster vaccination yielded modest but significant gains in protection against hospitalization and severe COVID-19 illness for 6 months after vaccination, but this protection did not persist beyond 6 months.

Following the roll-out of coronavirus disease 2019 (COVID-19) vaccines in 2021 and repeated Omicron waves in 2022, a large proportion of the world became vaccinated and infected, thus developing hybrid immunity. Although population-level immunity increased, COVID-19 caused more severe illness in the fall/winter of 2023–2024 than influenza [1]. As of 2024, national vaccination programs in the United States (US) put forth recommendations for the general adult population to receive annual variant-targeted COVID-19 and influenza vaccine doses in the fall; for older adults, a second COVID-19 vaccine dose is recommended approximately 6 months later [2]. In the summer of 2024, however, there was a large Omicron JN.1 wave, infecting many individuals who had received their variant-targeted XBB booster dose in the prior fall. These events prompted questions to determine how long variant-targeted booster-induced immunity protects against severe illness and if booster-vaccinated individuals with a severe acute respiratory syndrome coronavirus 2 (SARS-CoV-2) Omicron infection require their annual booster dose in the context of vaccine-derived and hybrid immunity.

The US Centers for Disease Control and Prevention recommends that an individual who has recently had COVID-19 may delay receiving the annual booster for 3 months. There are 2 main considerations to delay 3 months. First, risk of COVID-19 infection decreases following SARS-CoV-2 Omicron infection at least for 3–4 months [3]. Another study showed that postboost SARS-CoV-2 antibodies and B cells are muted by recent infection (<180 days) [4], providing evidence that receiving a booster within 3 months of an infection has diminished benefits. Yet, many adults consider delaying >3 months because they not only were recently infected but have also had a series of vaccine doses, including monovalent and variant-targeted booster doses, conferring hybrid immunity with multiple immune boosts.

Studies have shown that the protection conferred by hybrid immunity is longer lasting and more effective than protection conferred by vaccine- or infection-induced immunity alone [5–11]. Only 2 studies have assessed effectiveness against severe COVID-19 disease in a population with hybrid immunity; these were limited by short follow-up, imprecise estimates, and only up to 2–4 antigenic exposures (by either vaccination or infection) in populations studied before the era of variant-targeted booster formulations (before fall of 2022) [7, 11]. This evidence supports the hypothesis that more vaccine doses in a population with vaccine-derived and hybrid immunity will result in significant but incremental gains in protection against severe disease, even against viral variants with immune escape potential [12, 13].

This study evaluated the variant-targeted messenger RNA (mRNA) booster effectiveness against hospitalization or in-hospital severe illness due to COVID-19 pneumonia over a 12-month period, when BA.5, BQ.1/BQ.1.1, and XBB.1.5 Omicron lineage circulated, in a national cohort of booster-vaccinated US Veterans who had at least 3–5 antigenic exposures, and in a subpopulation after hybrid immunity.

## METHODS

### Ethics Statement

The institutional review board of the University of California, San Francisco, approved this study and waived requirement for consent as it involved secondary data.

### Study Design and Data Sources

We emulated a target randomized controlled trial of variant-targeted mRNA booster vaccination (bivalent vaccine targeting ancestral and Omicron BA.4/5, produced by either Moderna or Pfizer) compared with no variant-targeted vaccination for the prevention of hospitalization due to COVID-19 pneumonia and in-hospital severe illness among a booster-vaccinated population of US Veterans ≥18 years. The variant-targeted mRNA booster vaccine became available on 1 September 2022, marking the beginning of the eligibility period. Subsequently, we employed a sequential trial study design with propensity score matching (1:1) nested within a longitudinal, observational cohort (Supplementary Figure 1). For details about the target trial specification and protocol, see Supplementary Table 1.

We used data from the Veterans Health Administration (VHA) Corporate Data Warehouse (CDW) [14], COVID-19 Shared Data Resource [15], and Centers for Medicare and Medicaid Services to construct the adult cohort for this target trial emulation.

### Participants

Adults receiving care at VHA facilities were eligible for inclusion if they had a primary care visit from 1 July 2020 to 6 July 2022 and received at least 3 vaccine doses in the VA (initial primary series, followed by booster dose from 1 August 2021 to 31 August 2022), subsequently entering the period from 1 September 2022 onward when the variant-targeted booster dose became available [2]. Further inclusion and exclusion criteria are detailed in Supplementary Table 1. Many of these booster-vaccinated US Veterans also had a documented SARS-CoV-2 infection, so this population had at least 3–5 antigenic exposures (≥3 by booster dose from 1 August 2021 to 31 August 2022; ≥4 by variant-targeted booster dose from 1 September 2022 onward; ≥4–5 by vaccination plus infection).

### Matched Cohort

To conduct our nested sequential trial with propensity score matching, we created a series of weekly trials matching (1:1) those who received the variant-targeted booster dose to those who had not received the variant-targeted dose. To create a matched set, we estimated the propensity score by fitting a logistic model that regressed baseline and time-varying covariates on receipt of variant-targeted booster dose measured at the beginning of that week-specific trial. See Supplementary Table 2 for details of the covariates and matched cohort.

### Measurements

Treatment/exposure was receipt of the variant-targeted mRNA booster vaccination (bivalent vaccine targeting ancestral and Omicron BA.4/5), as extracted from the CDW. Those individuals who had documentation of a prior SARS-CoV-2 infection and receipt of booster vaccination were defined as having had preexisting hybrid immunity.

The 2 primary outcomes were (1) hospitalization due to COVID-19 pneumonia and (2) in-hospital severe COVID-19 illness. Hospitalization with COVID-19 pneumonia was defined as a diagnosis of COVID-19 pneumonia using *International Classification of Diseases, 10th Revision* (*ICD-10*) code J12.84 in the CDW [16], or documented by the clinical care team in the electronic medical record during hospitalization. In-hospital severe COVID-19 illness was defined as receiving ventilation, oxygen, or intubation (intensive care unit stay) during hospitalization due to COVID-19 pneumonia or death within 30 days after infection.

The outcome of COVID-19 pneumonia was verified through a combination of text processing–assisted chart review and *ICD-10* codes and has been described in more detail elsewhere [17]. Study staff reviewed 25% of charts in duplicate (95% agreement) and flagged unclear diagnoses of pneumonia (eg, emergency room note was only reference to pneumonia during hospitalization) for adjudication by 3 clinicians (J. D. K., S. K., D. M. B.).

For each person, follow-up started on day 7 after the variant-targeted booster dose (or for control subjects, the date of booster dose of the matched treated/exposed subject), assuming that the onset of protection is delayed by 7 days.

The follow-up period ended on the day of outcome of interest, death (>30 days after infection), or the end of the study period (31 August 2023), whichever happened first. The observation period included predominance of Omicron SARS-CoV-2 variants in the US, including the XBB sublineage [18].

### Statistical Analyses

We assessed the balance between treated/exposed subjects and control participants in the matched sample by computing standardized differences in the distribution of baseline covariates between the groups. In assessing associations in the overall cohort (population-level, vaccine-derived immunity), we matched treated/exposed and control participants on the logit of the propensity score, using a propensity score that incorporated time since last immunological event (defined as a prior booster dose or prior SARS-CoV-2 infection, whichever came later) in addition to other covariates. To assess impact in a population with hybrid immunity, we restricted the cohort to a subgroup of individuals with a documented SARS-CoV-2 infection (pre-Omicron, Omicron) and created a second matched set, using a propensity score that incorporated time since last booster dose in addition to other covariates. We found that standardized differences were all <0.1 after matching (Table 1, Supplementary Table 3).

**Table 1.** Characteristics of the Propensity Score–Matched Cohort

Characteristic	Total Cohort	Received Variant-Targeted Booster	Did Not Receive Variant-Targeted Booster Matched Pair	Standard Difference
No.	1 576 626	788 313	788 313	…
Sex				
Male	1 459 328 (92.6)	730 664 (92.7)	728 664 (92.4)	−0.0025
Female	117 298 (7.4)	57 649 (7.3)	59 649 (7.6)	0.0025
Age, y, mean (SD)	71.4 (11.1)	71.4 (11.1)	71.4 (11.1)	0.0000
Age group, y				
18–64	342 160 (21.7)	171 080 (21.7)	171 080 (21.7)	0.0000
65–74	561 310 (35.6)	280 655 (35.6)	280 655 (35.6)	0.0000
75–84	526 912 (33.4)	263 456 (33.4)	263 456 (33.4)	0.0000
≥85	146 244 (9.3)	73 122 (9.3)	73 122 (9.3)	0.0000
Race^a^				
American Indian or Alaska Native	10 110 (0.6)	4994 (0.6)	5116 (0.6)	0.0002
Asian	22 515 (1.4)	11 068 (1.4)	11 447 (1.5)	0.0005
Black or African American	296 281 (18.8)	148 564 (18.8)	147 717 (18.7)	−0.0011
Native Hawaiian or other Pacific Islander	12 668 (0.8)	6170 (0.8)	6498 (0.8)	0.0004
White	1 118 227 (70.9)	559 999 (71.0)	558 228 (70.8)	−0.0022
>1 race	11 411 (0.7)	5581 (0.7)	5830 (0.7)	0.0003
Missing	105 414 (6.7)	51 937 (6.6)	53 477 (6.8)	0.0020
Hispanic or Latino ethnicity^a^	94 617 (6.0)	46 213 (5.9)	48 404 (6.1)	0.0028
Currently married	961 632 (61.0)	482 726 (61.2)	478 906 (60.8)	−0.0048
Urban/rural^b^				
Highly rural or unknown	66 578 (4.2)	32 910 (4.2)	33 668 (4.3)	0.0010
Rural	416 285 (26.4)	208 181 (26.4)	208 104 (26.4)	−0.0001
Urban	1 093 763 (69.4)	547 222 (69.4)	546 541 (69.3)	−0.0009
BMI, kg/m^2^				
<18.5	10 457 (0.7)	5122 (0.6)	5335 (0.7)	0.0003
18.5–24.9	261 769 (16.6)	129 060 (16.4)	132 709 (16.8)	0.0046
25–29.9	546 479 (34.7)	273 796 (34.7)	272 683 (34.6)	−0.0014
≥30	643 828 (40.8)	324 431 (41.2)	319 397 (40.5)	−0.0064
Missing	114 093 (7.2)	55 904 (7.1)	58 189 (7.4)	0.0029
Comorbidities associated with severe COVID-19				
Hypertension	1 133 577 (71.9)	568 703 (72.1)	564 874 (71.7)	−0.0049
Diabetes	586 872 (37.2)	294 409 (37.3)	292 463 (37.1)	−0.0025
CKD				
CKD^c^	325 839 (20.7)	160 494 (20.4)	165 345 (21.0)	0.0062
No CKD	1 217 196 (77.2)	611 465 (77.6)	605 731 (76.8)	−0.0073
Severe CKD^d^	33 591 (2.1)	16 354 (2.1)	17 237 (2.2)	0.0011
Ischemic heart disease	377 199 (23.9)	187 073 (23.7)	190 126 (24.1)	0.0039
COPD/bronchiectasis	234 513 (14.9)	116 125 (14.7)	118 388 (15.0)	0.0029
Heart failure	144 094 (9.1)	70 433 (8.9)	73 661 (9.3)	0.0041
Immunocompromised^e^	99 400 (6.3)	48 603 (6.2)	50 797 (6.4)	0.0028
Stroke/TIA	68 172 (4.3)	33 478 (4.2)	34 694 (4.4)	0.0015
Dementia	42 757 (2.7)	20 467 (2.6)	22 290 (2.8)	0.0023
Cirrhosis	31 136 (2.0)	15 597 (2.0)	15 539 (2.0)	−0.0001
Cancer, including lymphoma and leukemia^f^	26 921 (1.7)	13 109 (1.7)	13 812 (1.8)	0.0009
ESRD on dialysis	11 595 (0.7)	5677 (0.7)	5918 (0.8)	0.0003
Cancer other^f^	18 462 (1.2)	9677 (1.2)	8785 (1.1)	−0.0011
Spinal cord injury	9505 (0.6)	4653 (0.6)	4852 (0.6)	0.0003
Social and behavioral risk factors				
Current smoker	277 076 (17.6)	137 366 (17.4)	139 710 (17.7)	0.0030
Alcohol abuse^g^	114 276 (7.2)	56 722 (7.2)	57 554 (7.3)	0.0011
Substance use^h^	81 442 (5.2)	40 416 (5.1)	41 026 (5.2)	0.0008
Housing problems^i^	58 105 (3.7)	28 810 (3.7)	29 295 (3.7)	0.0006
VA priority^j^				
1	650 821 (41.3)	326 620 (41.4)	324 201 (41.1)	−0.0031
2	111 347 (7.1)	55 740 (7.1)	55 607 (7.1)	−0.0002
3	219 430 (13.9)	110 538 (14.0)	108 892 (13.8)	−0.0021
4	15 210 (1.0)	7259 (0.9)	7951 (1.0)	0.0009
5	230 107 (14.6)	114 181 (14.5)	115 926 (14.7)	0.0022
6	70 958 (4.5)	36 189 (4.6)	34 769 (4.4)	−0.0018
7	67 662 (4.3)	33 646 (4.3)	34 016 (4.3)	0.0005
8	210 201 (13.3)	103 683 (13.2)	106 518 (13.5)	0.0036
Missing	890 (0.1)	457 (0.1)	433 (0.1)	0.0000
CAN score^k^				
0–24.9	129 116 (8.2)	61 522 (7.8)	67 594 (8.6)	0.0077
25–49.9	349 990 (22.2)	177 873 (22.6)	172 117 (21.8)	−0.0073
50–74.9	542 280 (34.4)	277 011 (35.1)	265 269 (33.7)	−0.0149
75–100	535 393 (34.0)	262 017 (33.2)	273 376 (34.7)	0.0144
Missing	19 847 (1.3)	9890 (1.3)	9957 (1.3)	0.0001
Home-based primary care	24 687 (1.6)	11 640 (1.5)	13 047 (1.7)	0.0018
Last documented SARS-CoV-2 infection^l^				
Pre-Omicron	109 872 (7.0)	54 936 (7.0)	54 936 (7.0)	0.0000
Omicron	83 080 (5.3)	41 540 (5.3)	41 540 (5.3)	0.0000
No prior infection	1 383 674 (87.8)	691 837 (87.8)	691 837 (87.8)	0.0000
Timing of variant-targeted booster dose				
1 Sep–15 Dec 2022	…	672 277 (85.3)	…	…
After 15 Dec 2022	…	116 036 (14.7)	…	…

Data are presented as No. (%) unless otherwise indicated. Propensity score includes time since last booster dose. All standardized differences were <0.1 after matching.

Abbreviations: BMI, body mass index; CAN, Care Assessment Need; CHF, chronic heart failure; CKD, chronic kidney disease; COPD, chronic obstructive pulmonary disease; COVID-19, coronavirus disease 2019; ESRD, end-stage renal disease; SARS-CoV-2, severe acute respiratory syndrome coronavirus 2; SD, standard deviation; TIA, transient ischemic attack; VA, Veterans Affairs.

^a^Race/ethnicity was assessed using self-identified data found in Veteran health records.

^b^Urban/rural status was assessed using defined based on the Rural Urban Commuting Area categories developed by the Department of Agriculture and the Department of Health and Human Services' Health Resource and Services Administration.

^c^CKD defined as having a glomerular filtration rate between 30 and 60 mL/min/1.73 m^2^.

^d^Severe CKD defined as having a glomerular filtration rate <30 mL/min/1.73 m^2^.

^e^Immunocompromised definition based on medications and history of cancer (see Supplementary Table 5 for list of medications).

^f^Cancer definition based on 1 inpatient or 2 outpatient diagnosis codes in the VA (see Supplementary Table 2).

^g^Alcohol use defined as 1 outpatient or 1 inpatient code within 2 years of index date.

^h^Including cannabis, opioids, inhalants.

^i^Housing problems defined as homelessness, inadequate housing, and other problems related to housing and economic circumstances.

^j^VA defined based on factors including military service history, disability rating, and income level to identify Veterans to determine enrollment priority; score ranges from 1 to 8 with 1 being the highest priority. This is a surrogate for socioeconomic status.

^k^The CAN score is a predictive analytic tool that estimates the relative probability of hospitalization and death within 90 days or 1 year from the calculation date. The Office of Clinical Systems Development and Evaluation (10E2A) produces the weekly CAN score report to help identify the highest-risk patients in a primary care panel or a cohort. We used the 1-year score.

^l^Last documented infection occurred 91 or more days prior to booster shot.

To estimate the association between bivalent vaccination and the 2 outcomes, we fit univariate Cox regression models in the matched sample. We used a robust variance estimator to account for the matched nature of the sample [19]. We first fit Cox models on the overall matched sample for each of the 2 outcomes, controlling for time since last immunological event in the propensity score, and used the estimated hazard ratio (HR) to compute the relative vaccine effectiveness (rVE = [1 − HR] * 100%) [20, 21]. We then stratified the sample by period of the most recent documented SARS-CoV-2 infection (pre-Omicron, Omicron), fit Cox models with robust standard errors to the matched sample controlling for time since last booster dose in the propensity score, and estimated the association for each outcome over each of these 3 groups.

We fit time-stratified Cox models, relaxing the proportional hazards assumption of the Cox model (ie, a constant HR over follow-up time). These models estimated separate HRs for different periods of follow-up time (0–6 months and 6–12 months after baseline for analyses stratified by period of most recent SARS-CoV-2 infection and 0–3 months, 3–6 months, 6–9 months, and 9–12 months after baseline for overall analyses). Finally, to evaluate for residual confounding, we repeated the analysis in the overall cohort with the negative control outcome of hospitalization over 0–7 days, given the assumption of similar risk between groups until booster-induced protection starting at day 7 (Supplementary Figure 2). All analyses were conducted in R version 4.2.1, including the “survival” package.

## RESULTS

From an initial group of 6 286 624 participants, the matched cohort consisted of 1 576 626 participants; all of them either did or did not receive the bivalent booster dose (Table 1, Supplementary Table 3, Supplementary Figure 3). Among these participants, 78.3% were age >65 years, and 92.6% were male; 12.3% had a history of SARS-CoV-2 infection, including 7.0% infected in the pre-Omicron era and 5.3% in the Omicron era. The largest amount of missingness (7.2%) occurred with the body mass index variable.

Of 788 313 participants who received the booster, 672 277 (85.3%) received a dose between 1 September and 15 December 2022 (Omicron sublineage predominant variant period prior to XBB) and 116 036 (14.7%) received a dose between 15 December 2022 and 28 February 2023 (Omicron XBB predominant variant period).

### Effectiveness of a Variant-Targeted Booster Dose in the Overall Population With Vaccine-Derived Immunity

**Figure 1.**
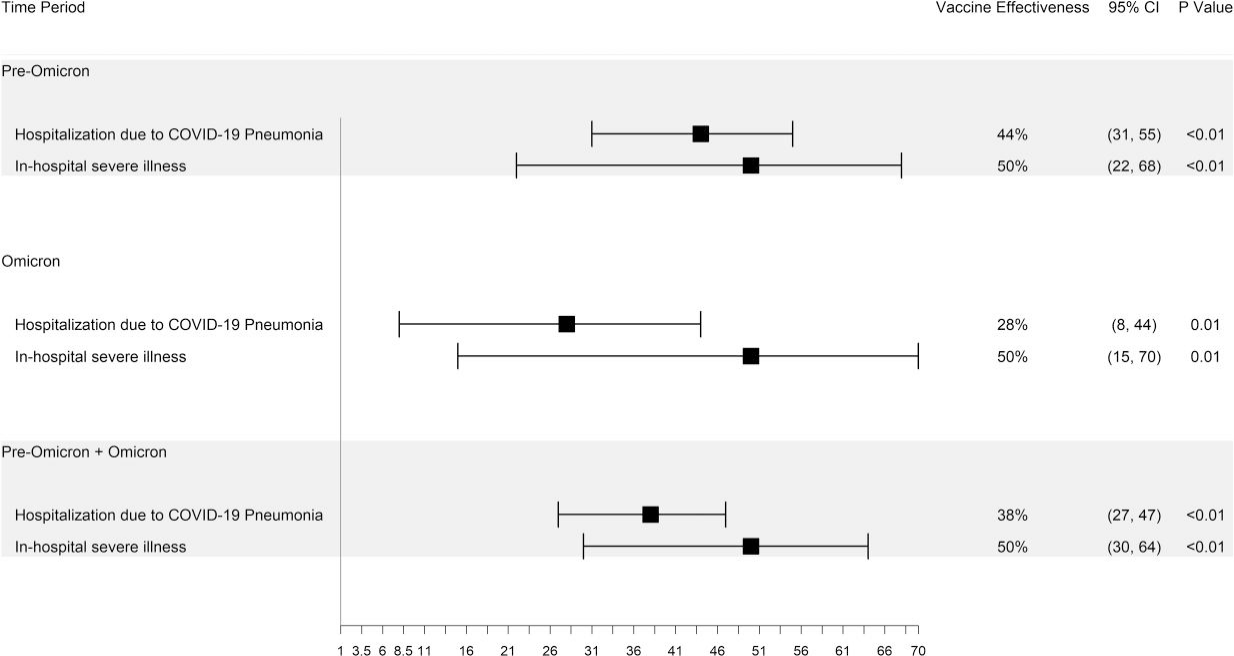
Relative vaccine effectiveness in the overall cohort population (vaccine-derived immunity) after a variant-targeted booster dose against hospitalization due to COVID-19 pneumonia and in-hospital severe illness. The comparator group is a booster-vaccinated group without receipt of a variant-targeted booster dose. These include time-fixed and time-segmented analyses. Abbreviations: CI, confidence interval; COVID-19, coronavirus disease 2019.

In the overall cohort, we found that variant-targeted booster effectiveness prevented hospitalization and in-hospital severe illness. Compared to the group without a booster dose, those with booster dose had an rVE of 29% for hospitalization due to COVID-19 pneumonia (95% confidence interval [CI], 25%–34%) and had rVE estimates of 25% for in-hospital severe illness (95% CI, 15%–33%). Estimates of rVE against hospitalization were most protective during the initial 3-month period (rVE, 45% [95% CI, 40%–50%]), followed by a waning but significantly protective effectiveness (rVE: 18% [95% CI, 9%–26%]) during the second 3-month period (3–6 months), subsequently trending to the null value (6–9 months, 9–12 months). There was a similar pattern for in-hospital severe illness (Figure 1).

### Effectiveness of Variant-Targeted Booster Dose After Hybrid Immunity

**Figure 2.**
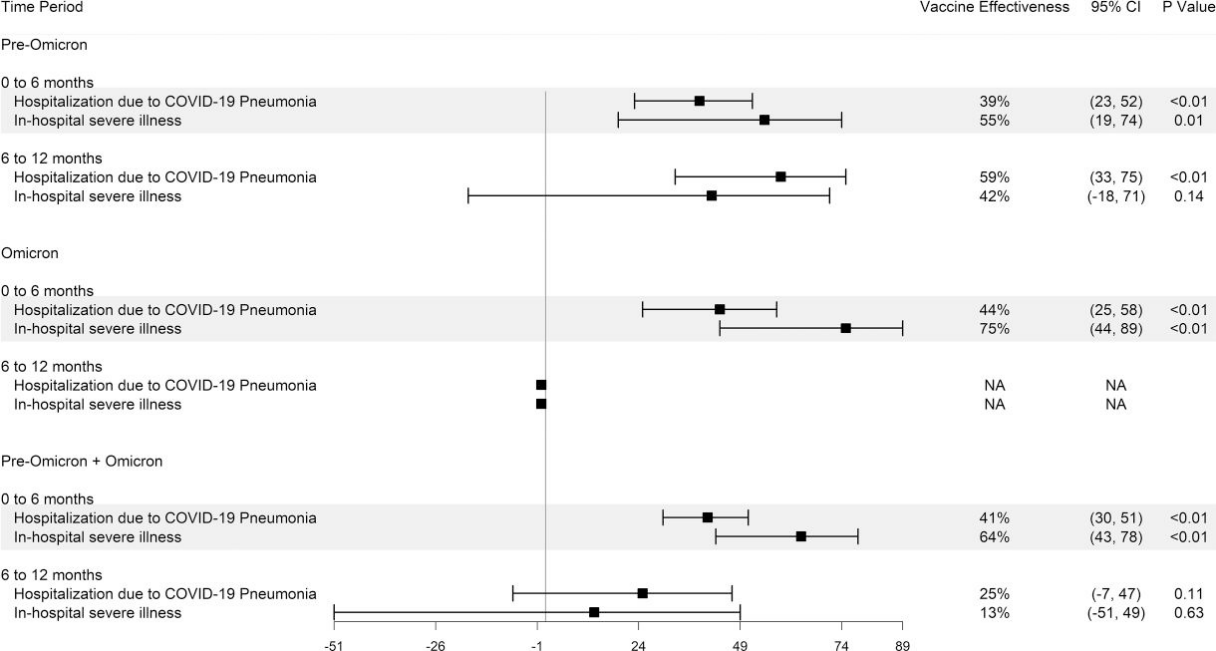
Relative vaccine effectiveness in population with hybrid immunity after a variant-targeted booster dose against hospitalization due to COVID-19 pneumonia and in-hospital severe illness, stratified by period of prior infection (time-fixed). The comparator group is a booster-vaccinated group without receipt of a variant-targeted booster dose. Abbreviations: CI, confidence interval; COVID-19, coronavirus disease 2019.

In the cohort with hybrid immunity (infected by pre-Omicron or Omicron variants), rVE estimates were 38% for hospitalization (95% CI, 27%–47%) and 50% for in-hospital severe illness (95% CI, 30%–64%). Within this cohort, we evaluated the impact of the variant-targeted booster dose on the clinical outcomes by last documented infection (pre-Omicron era, Omicron era). In the pre-Omicron-infected subgroup, rVE estimates were 44% for hospitalization (95% CI, 31%–55%) and 50% for in-hospital severe illness (95% CI, 22%–68%). In the Omicron-infected subgroup, rVE estimates were 28% for hospitalization (95% CI, 8%–44%) and 50% for in-hospital severe illness (95% CI, 15%–70%) (Figure 2). Although rVE for hospitalization were lower in the Omicron-infected subgroup compared with the pre-Omicron subgroup, our assessment of heterogeneity did not reveal evidence of interaction (hospitalization: interaction *P* = .34; severe illness: interaction *P* = .44).

### Subgroup Analyses of Those With Hybrid Immunity Over 6-Month Intervals

When evaluating hospitalization during the initial 6-month period, rVE estimates were 41% (95% CI, 30%–51%) in the hybrid immunity group, 39% (95% CI, 23%–52%) in the pre-Omicron-infected subgroup, and 44% (95% CI, 25%–58%) in the Omicron-infected subgroup. During this initial 6-month period, we assessed rVE estimates of hospitalization for heterogeneity in effects by pre-Omicron and Omicron subgroups and found no evidence of interaction (interaction *P* = .66). These groups experienced a greater protective benefit against in-hospital severe illness during the same 6-month period. Relative VE estimates for severe illness were 64% (95% CI, 43%–78%) in the hybrid immunity group, 55% (95% CI, 19%–74%) in the pre-Omicron-infected subgroup, and 75% (95% CI, 44%–89%) in the Omicron-infected subgroup. There was also no evidence of interaction (*P* = .20).

**Figure 3.**
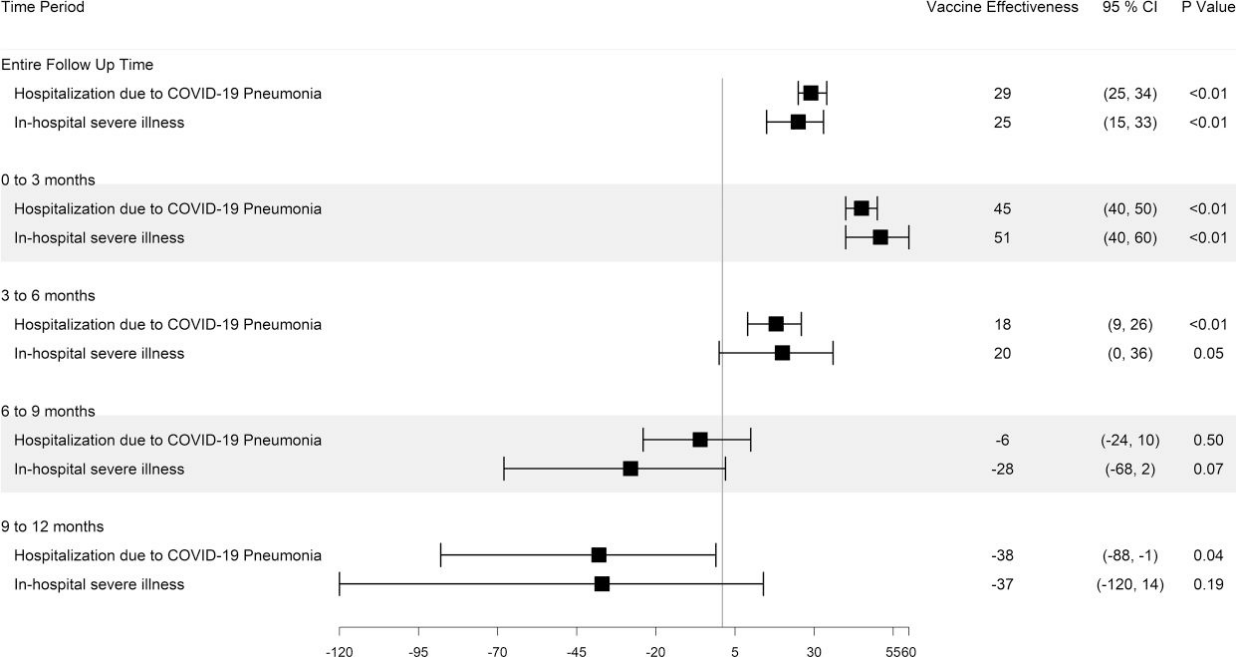
Relative vaccine effectiveness in population with hybrid immunity after a variant-targeted booster dose against hospitalization due to COVID-19 pneumonia and in-hospital severe illness, stratified by period of prior infection (time-segmented). The comparator group is a booster-vaccinated group without receipt of a variant-targeted booster dose. Abbreviations: CI, confidence interval; COVID-19, coronavirus disease 2019; NA, not applicable.

During the second 6-month period (6–12 months), the variant-targeted booster dose conferred a sustained benefit against hospitalization in the pre-Omicron subgroup (rVE, 59% [95% CI, 33%–75%]); this benefit attenuated when assessed in the combined pre-Omicron and Omicron infected subgroups (cohort with hybrid immunity) (rVE, 25% [95% CI, −7% to 47%]). We had insufficient power to evaluate the second 6-month period in the Omicron-infected group, but we observed how combining these data with the pre-Omicron group drove the attenuation. Estimates of rVE for in-hospital severe illness included the null value for groups with hybrid immunity (combined) and with pre-Omicron infection only (Figure 3).

### Effectiveness of Booster Targeting Ancestral and Omicron BA.4/5 Lineages During the Viral Variant Era of XBB

We stratified analyses for individuals who received a booster dose before and after the period when XBB became the predominant viral variant. In the overall cohort, the booster dose was found to be protective against hospitalization during the pre-XBB and post-XBB era for hospitalization due to COVID-19 pneumonia. Compared to those who did not receive the booster dose, those who received the booster dose had an rVE of 30% for hospitalization due to COVID-19 pneumonia during the pre-XBB era (95% CI, 25%–34%) and had an rVE of 32% for hospitalization due to COVID-19 pneumonia during the XBB era (95% CI, 14%–46%). For in-hospital severe illness, the booster dose was protective during the pre-XBB era with an rVE of 32% (95% CI, 22%–40%), but not during the post-XBB era (VE, 17% [95% CI, −22% to 43%]). These findings were similar when we restricted to the population with hybrid immunity (Supplementary Table 4).

## DISCUSSION

These national US cohorts of adults with vaccine-derived and hybrid immunity had significant gains in protection from the variant-targeted mRNA booster dose against hospitalization and in-hospital severe illness due to COVID-19 pneumonia. In both cohorts (vaccine-derived and hybrid immunity), these gains in protection generally waned after 6 months postvaccination, except for those infected in pre-Omicron era who had sustained benefit over 6–12 months. Gains in protection from the booster dose were observed during the pre-XBB and XBB variant eras, suggesting that additional booster doses may have ongoing benefits even when viral variants emerge with well-known immune escape potential.

This study supports current recommendations from national vaccine programs that individuals would benefit from an annual updated vaccine dose following their last dose. There continues to be a need to balance time-limited gains in protection (6 months) with a pragmatic approach (annual dosing schedules), except for high-risk populations in which more frequent dosing is recommended. This study also demonstrates that when SARS-CoV-2 emerges into an immune evasive variant as determined by laboratory testing, a booster dose (eg, against the pre-XBB variant) is still likely to confer protection against that subsequent viral variant (eg, against XBB variant).

Over the pandemic, in studies of rVE, the comparator group has changed to consider more immunological events, either infection or vaccine doses, to be more consistent with the current experience of the majority of the population. Based on a few, limited studies using a comparator group with hybrid immunity from more immunological events, gains from relative booster effectiveness may be incremental in magnitude and short in duration. Our study supports emerging evidence of modest but significant benefits from boosters, adding to a sparse evidence base about hybrid immunity after greater versus fewer doses of vaccine.

This study has several limitations. As an observational study, there is risk of residual confounding. However, the use of negative control checks may relax these concerns. In the era of Omicron predominant sublineages, use of at-home testing has been common, so underreporting of documented infections is likely, particularly in the group without any history of a prior SARS-CoV-2 infection. By focusing on the cohort with hybrid immunity, assessments for heterogeneity by those with an infection from pre-Omicron versus Omicron variants were less likely to have measurement bias because they represented the proportion of the population who seek diagnostic testing at healthcare facilities when sick. In general, we selected a population that actively received their primary care at VHA facilities and combined multiple data sources (Medicare and Veterans Affairs data) to prevent detection bias. The Veteran population, however, is not representative of the general US population, particularly because of its relatively smaller female and Latino populations. External validity to younger populations should also be approached with caution given that the Veteran population is older with a significant burden of comorbidities. The older Veteran population, on the other hand, allowed for sufficient events to power analyses of relative booster effectiveness against hospitalization and in-hospital severe illness due to COVID-19 pneumonia.

In these cohorts with vaccine-derived and hybrid immunity, we conclude that the variant-targeted booster dose conferred modest gains in protection against hospitalization and in-hospital severe illness due to COVID-19 pneumonia for 6 months after vaccination, even when a variant of SARS-CoV-2 emerges with immune evasive potential. Vaccine-induced protection did not persist beyond 6 months. This report underscores the importance of developing SARS-CoV-2 vaccines with mRNA and alternative delivery strategies that can generate durable, broadly protective immune response against severe disease outcomes.

## Supplementary Material

ciaf124_Supplementary_Data

## Supplementary Data

Supplementary materials are available at *Clinical Infectious Diseases* online. Consisting of data provided by the authors to benefit the reader, the posted materials are not copyedited and are the sole responsibility of the authors, so questions or comments should be addressed to the corresponding author.

## Notes

***Author contributions*.** J. D. K., K. J. H., N. C. L., W. J. B., P. C. T., P. C. A., D. M. B., and S. K. conceived and designed the study. J. D. K., K. J. H., S. L., E. N. L., H. S. K., C. C. A., D. M. B., and S. K. contributed to data collection and curation. J. D. K., K. J. H., S. L., E. N. L., C. C. A., P. C. A., D. M. B., and S. K. accessed and verified all data, did the data analysis, and drafted the first version of the manuscript. J. D. K., K. J. H., N. C. L., W. J. B., C. C. A., A. L. B., P. C. T., P. C. A., D. M. B., and S. K. revised the manuscript. All authors approved the final version.

***Acknowledgments*.** The authors thank the US Veterans who received vaccine booster doses and contributed data to our study. They appreciate our San Francisco–based chart review team and other Veterans Health Administration employees who have supported aspects of this study.

***Data availability*.** Data to generate the findings of this study are available from the US Department of Veterans Affairs. VA data are made freely available to researchers behind the VA firewall through the VA Informatics and Computing Infrastructure (VINCI) and with an approved VA study protocol. More information is available at https://www.virec.research.va.gov.

***Disclaimer.*** The sponsor had no role in any of these aspects of the study, including the decision to submit for publication, right to veto publication, or right to control the decision regarding to which journal the manuscript was submitted.

***Financial support***. This work was supported by the Department of Veterans Affairs Clinical Science Research and Development (I01 grant number CX002417 to J. D. K. and S. K.) and the National Institute of Allergy and Infectious Diseases (NIAID) (K23 grant number AI146268 to J. D. K.). N. C. L. is supported by a NIAID New Innovator Award (DP2 AI170485).

## References

[1] Xie Y, Choi T, Al-Aly Z. Mortality in patients hospitalized for COVID-19 vs influenza in fall-winter 2023–2024. JAMA 2024; 331:1963–5. 38748411 10.1001/jama.2024.7395 PMC11097092

[2] Centers for Disease Control and Prevention . COVID-19 vaccine boosters. 2022. Available at: https://www.cdc.gov/coronavirus/2019-ncov/vaccines/booster-shot.html. Accessed 30 March 2025.

[3] COVID-19 Forecasting Team . Past SARS-CoV-2 infection protection against re-infection: a systematic review and meta-analysis. Lancet 2023; 401:833–42. 36930674 10.1016/S0140-6736(22)02465-5 PMC9998097

[4] Buckner CM, Kardava L, El Merhebi O, et al Interval between prior SARS-CoV-2 infection and booster vaccination impacts magnitude and quality of antibody and B cell responses. Cell 2022; 185:4333–46.e14. 36257313 10.1016/j.cell.2022.09.032 PMC9513331

[5] Bobrovitz N, Ware H, Ma X, et al Protective effectiveness of previous SARS-CoV-2 infection and hybrid immunity against the Omicron variant and severe disease: a systematic review and meta-regression. Lancet Infect Dis 2023; 23:556–67. 36681084 10.1016/S1473-3099(22)00801-5 PMC10014083

[6] Nealon J, Mefsin YM, McMenamin ME, Ainslie KEC, Cowling BJ. Reported effectiveness of COVID-19 monovalent booster vaccines and hybrid immunity against mild and severe Omicron disease in adults: a systematic review and meta-regression analysis. Vaccine X 2024; 17:100451. 38379667 10.1016/j.jvacx.2024.100451 PMC10877401

[7] Carazo S, Skowronski DM, Brisson M, et al Effectiveness of previous infection-induced and vaccine-induced protection against hospitalisation due to Omicron BA subvariants in older adults: a test-negative, case-control study in Quebec, Canada. Lancet Healthy Longev 2023; 4:e409–20. 37459879 10.1016/S2666-7568(23)00099-5

[8] Shrestha NK, Shrestha P, Burke PC, Nowacki AS, Terpeluk P, Gordon SM. Coronavirus disease 2019 vaccine boosting in previously infected or vaccinated individuals. Clin Infect Dis 2022; 75:2169–77. 35476018 10.1093/cid/ciac327 PMC9129118

[9] Nielsen KF, Moustsen-Helms IR, Schelde AB, et al Vaccine effectiveness against SARS-CoV-2 reinfection during periods of Alpha, Delta, or Omicron dominance: a Danish nationwide study. PLoS Med 2022; 19:e1004037. 36413551 10.1371/journal.pmed.1004037 PMC9681105

[10] Plumb ID, Feldstein LR, Barkley E, et al Effectiveness of COVID-19 mRNA vaccination in preventing COVID-19–associated hospitalization among adults with previous SARS-CoV-2 infection—United States, June 2021–February 2022. MMWR Morb Mortal Wkly Rep 2022; 71:549–55. 35421077 10.15585/mmwr.mm7115e2 PMC9020856

[11] Wu S, Li Y, Baral S, et al Protection of prior SARS-CoV-2 infection, COVID-19 boosters, and hybrid immunity against Omicron severe illness: a population-based cohort study of five million residents in Canada. PLoS One 2024; 19:e0299304. 38394091 10.1371/journal.pone.0299304 PMC10889649

[12] Pilz S, Ioannidis JPA. Does natural and hybrid immunity obviate the need for frequent vaccine boosters against SARS-CoV-2 in the endemic phase? Eur J Clin Invest 2023; 53:e13906. 36366946 10.1111/eci.13906 PMC9878177

[13] Pilz S, Theiler-Schwetz V, Trummer C, Krause R, Ioannidis JPA. SARS-CoV-2 reinfections: overview of efficacy and duration of natural and hybrid immunity. Environ Res 2022; 209:112911. 35149106 10.1016/j.envres.2022.112911 PMC8824301

[14] US Department of Veterans Affairs . VA informatics and computing infrastructure, Corporate Data Warehouse (CDW), Health Services Research and Development. Available at: https://www.hsrd.research.va.gov/for_researchers/vinci/cdw.cfm. Accessed 30 March 2025.

[15] DuVall S, Scehnet J. Introduction to the VA COVID-19 shared data resource and its use for research. 2020. Available at: https://www.hsrd.research.va.gov/cyberseminars/catalog-upcoming-session.cfm?UID=3810. Accessed 30 March 2025.

[16] US Department of Veterans Affairs . Corporate Data Warehouse. Available at: https://www.datahub.va.gov/dataset/Corporate-Data-Warehouse-CDW-/ftpi-epf7/about_data. Accessed 30 March 2025.

[17] Kelly JD, Leonard S, Hoggatt KJ, et al Incidence of severe COVID-19 illness following vaccination and booster with BNT162b2, mRNA-1273, and Ad26.COV2.S vaccines. JAMA 2022; 328:1427–37. 36156706 10.1001/jama.2022.17985 PMC9513709

[18] The New York Times . Coronavirus in the U.S.: latest map and case count. 2023. Available at: https://www.nytimes.com/interactive/2021/us/covid-cases.html. Accessed 30 March 2025.

[19] Austin PC . The performance of different propensity score methods for estimating marginal hazard ratios. Stat Med 2013; 32:2837–49. 23239115 10.1002/sim.5705 PMC3747460

[20] Halloran ME, Longini IM, Struchiner CJ. Design and interpretation of vaccine field studies. Epidemiol Rev 1999; 21:73–88. 10520474 10.1093/oxfordjournals.epirev.a017990

[21] Ioannidis JPA . Estimating conditional vaccine effectiveness. Eur J Epidemiol 2022; 37:885–90. 36155868 10.1007/s10654-022-00911-3 PMC9510183

