# Supplementary material for: Annual Variant-Targeted Vaccination to Prevent Severe COVID-19 in Cohorts With Vaccine-Derived and Hybrid Immunity
Source: Clin Infect Dis. 2025 Mar 14;81(2):222–30. doi: 10.1093/cid/ciaf124 (PMC12448581; doi:10.1093/cid/ciaf124)
Supplement: ciaf124_Supplementary_Data [file ciaf124_supplementary_data.docx]

**Supplemental Material**

**Annual Variant-Targeted Vaccination to Prevent Severe Coronavirus Disease 2019 in Cohorts with Vaccine-Derived and Hybrid Immunity**

**Authors:**

J. Daniel Kelly^1,2,3,4*^, MD PhD

Katherine J. Hoggatt^1,2^, PhD

Nathan C. Lo^5^, MD PhD

Samuel Leonard^1^, MS

W. John Boscardin^3^, PhD

Hye Sun Kim^1^, MPH

Emily N. Lum^1^, MPH

Charles C. Austin^1, 6,7^, MDiv

Amy L. Byers^1,8^, PhD

Phyllis C. Tien^1,2^, MD

Peter C. Austin^9^, PhD

Dawn M. Bravata^6,7,11^, MD

Salomeh Keyhani^1,2^, MD

**Affiliations:**

1. Center for Data to Discovery and Delivery Innovation (3DI), San Francisco VA Medical Center, San Francisco, CA, USA;

2. Department of Medicine, University of California, San Francisco (UCSF), CA, USA;

3. Department of Epidemiology and Biostatistics, UCSF, CA, USA;

4. F.I. Proctor Foundation, UCSF, CA, USA;

5. Division of Infectious Diseases and Geographic Medicine, Department of Medicine, Stanford University, Stanford, CA, USA;

6. Department of Veterans Affairs (VA) Health Services and Development (HSR&D) Center for Health Information and Communication (CHIC), Richard L. Roudebush VA Medical Center; Indianapolis

7. Department of Medicine, Richard L. Roudebush VA Medical Center, Indianapolis, IN, USA; 8. Department of Psychiatry, Weill Institute for Neurosciences, UCSF, CA, USA;

9. Cardiovascular Research Program, Institute for Clinical Evaluative Sciences, Toronto, Ontario, Canada;

10. Department of Medicine, Indiana University School of Medicine, Indianapolis, IN, USA;

11. William M. Tierney Center for Health Services Research, Regenstrief Institute, Indianapolis, IN, USA.

***Corresponding author contact information:** J. Daniel Kelly, MD, PhD; 550 16^th^ St., San Francisco, CA 94143; phone: (415) 502-3192; fax: (415) 476-9364; email: [dan.kelly@ucsf.edu](mailto:dan.kelly@ucsf.edu); Twitter: @DanKelly_MD

**Supplemental Material**

**Annual Variant-Targeted Vaccination to Prevent Severe Coronavirus Disease 2019 in Cohorts With Vaccine-Derived and Hybrid Immunity**

**INDEX**

1. SFigure 1 Nested sequential trial study design with matching.
2. STable 1 Target trial protocol with trial specifications and emulation.
3. STable 2 Description of methods for matched cohort. Variable definitions and covariates

included in the propensity score model.

1. STable 3 Characteristics of propensity-score matched cohort. Propensity score includes time since last immunological event. All standardized differences were all <0.1 after matching.
2. SFigure 2 Kaplan-Meier survival curve of hospitalization 0 to 7 days after vaccination to

evaluate the negative outcome control. Survival probabilities were similar between groups.

1. SFigure 3 Flow Diagram.
2. STable 4ab Relative vaccine effectiveness (VE) in the overall cohort (STable 4a) and in

population with hybrid immunity (STable 4b) against hospitalization due to COVID-19 pneumonia and in-hospital severe illness, stratified analyses by receipt of the booster dose before and during the XBB Omicron variant predominant era. Patients vaccinated before January 1, 2022, were part of pre-XBB era, and those vaccinated after January 1, 2022 were part of XBB era. These VE estimates represent propensity-score matched estimates from two cohorts (pre-XBB, XBB era) and followed until the end of the era under observation.

1. STable 5 Immunocompromised status defined based on receipt of three classes of drugs

**SFigure 1. Nested sequential trial study design with matching**


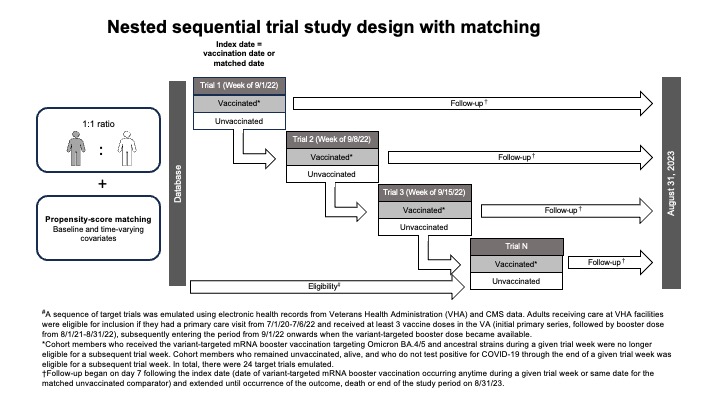


**STable 1. Target trial protocol with trial specification and emulation. Target trial specification describes the ideal study that would have been conducted as a randomized controlled trial. Target trial emulation explicitly reports how we used observational data in an attempt to replicate the randomized controlled trial. This table uses a framework that involves first specifying the protocol of the target trial and then emulating the trial as closely as possible using observation data.**

| **Protocol component** | **Target trial specification** | **Target trial emulation** |
| --- | --- | --- |
| Eligibility criteria | - Aged > 18 years between July 1, 2020 – July 6, 2022. - Had a primary care visit at a VHA facility between 2020 and 2022. - Documented receipt of all U.S. FDA authorized doses of the initial vaccination series of an mRNA vaccine and subsequently had documented receipt of at least one booster dose before 9/1/2022. - Did not receive a combination of mRNA and viral vector doses. - Not in nursing home between 9/1/20-9/1/22. - Not in hospice care between 9/1/20-9/1/22. - Did not have COVID-19 infection or diagnosis within 90 days before 9/2022. | Same as for the target trial, except:   - We identified previous documented SARS-CoV-2 infections using the ICD-10 codes given at the VA, Medicare, Medicaid, or VA COVID-19 labs. - Data on the listed allergic reactions are not consistently available for all Veterans, but we assumed that receiving the vaccine indicates there was a determination of no previous allergic reaction. |
| Treatment strategies | Receipt of the mRNA booster dose (either bivalent BNT162b2 vaccine or mRNA-1273 vaccine) | Same as for the target trial. We defined the date of vaccination using VA, Medicare, or Medicaid records in both the *immunization* domain and procedures recorded in the *Outpatient* or *Inpatient* domains. |
| Treatment assignment | Eligible participants are randomly assigned to receive the mRNA booster dose or no dose. | *Propensity-score matching:*  We assumed random assignment after matching individuals who were vaccinated with the booster dose in a 1:1 ratio to eligible individuals who did not receive the booster dose, using the following matching factors:   1. Week of booster dose 2. Propensity score estimated from baseline covariates and a time-varying covariate (time since last booster dose or immunological event)   For each patient who received the booster dose, we found a nearest neighbor match based on the propensity score, with exact matching on age. We assembled the weekly risk sets into the final matched dataset for subsequent analysis. |
| Outcomes | - Hospitalization due to COVID 19 pneumonia (hospitalization defined as a diagnosis of COVID-19 pneumonia using ICD-10 code J12.84 or documented by the clinical care team in the electronic medical record during hospitalization). - In-hospital severe illness (defined as being hospitalized due to COVID-19 pneumonia and having an ICU stay and/or death) | Same as for the target trial. |
| Follow-up | For each person, follow-up started on the day of bivalent booster dose and ended on the day of the outcome of interest, death, or the end of the study period (8/31/2023), whichever happened first. | Same as for the target trial. |
| Casual contrasts | Intention-to-treat effect  Per-protocol effect, i.e., the average effect treatment on the treated group | Observational analogue of the per-protocol effect. |
| Statistical analysis | Estimates of hazard ratios and relative vaccine effectiveness comparing those with and without the booster dose.  Interaction analyses by period of most recent documented SARS-CoV-2 infection. Time-varying Cox models that estimated separate HRs for different periods of follow up time. | Same as for the target trial. |

**STable 2. Description of methods for matched cohort. Variable definitions and covariates included in the propensity score model**

*Matched cohort*

To conduct our nested sequential trial with propensity-score matching, we created a series of weekly trials matching (1:1) those who received the variant-targeted booster dose to those who had not received the variant-targeted dose. For every week during the variant-targeted booster period, starting 9/1/22, we identified patients who were at risk for the outcome, inclusive of those who had and had not received the booster dose. To create a matched set, we estimated the propensity score by fitting a logistic model that regressed baseline and time-varying covariates on receipt of variant-targeted booster dose measured at the beginning of that week-specific trial. See Supplement for a detailed description of the covariates and their use in analyses (**STable 2**). For each patient who received the variant-targeted booster dose, we found a nearest neighbor match of a patient who did not receive the variant-targeted booster dose based on the propensity score. We fit a propensity score model for each week. For each of these weekly risk sets, patients who had the variant-targeted booster dose at a later date were eligible to be selected into the matched set prior to the vaccination date. Each patient could be selected into the unexposed arm in subsequent weekly trials. We assembled the weekly risk sets into the final matched dataset for subsequent analysis. Each patient in the exposed and unexposed groups of the cohort was followed until the date of the outcome or censoring (on the date of death or the end of follow-up, whichever came first). For matched sets where the matched control subject went on to receive the variant-targeted booster dose, the matched set was censored on the date the control subject received the booster dose. Missing data were handled as a ‘missing’ category for the variable.

*Variable definitions and covariates included in the propensity score model*

Covariates were age, sex, race, ethnicity, marital status, urban-rural residence, body mass index (BMI), co-morbid conditions, receipt of home-based primary care, calendar period associated with SARS-CoV-2 predominant variant (pre-Omicron era, Omicron era, XBB era), time since receipt of last booster dose (prior to baseline), or time since last immunological event (defined as last occurrence either of booster dose or SARS-CoV-2 infection prior to baseline). Race and ethnicity have been associated with adverse COVID-19 outcomes in other populations; acquisition of race/ethnicity data in VHA occurred from patient or proxy self-report based on pre-specified categories defined in VHA Handbook 1601A.01.

Comorbid conditions associated with poor COVID-19 clinical outcomes in the literature (hospitalization and mortality) included hypertension, heart failure, ischemic heart disease, diabetes, stroke or transient ischemic attack, chronic obstructive pulmonary disease or bronchiectasis, cirrhosis, dementia, spinal cord injury, immunocompromised status in the past one year, any chronic kidney disease (CKD), severe CKD, dialysis, and cancer (solid organ, hematologic malignancies [lymphoma/leukemia], other). Behavioral risk factors were current smoking, alcohol use disorder, and any non-alcohol- or non-smoking-related substance use disorder. Social risk factors were housing problems (use of housing services in past year) and Veteran priority score (surrogate for income status). Reinfection was defined as two SARS-CoV-2 test results and/or COVID-19 diagnoses greater than 90 days apart.

The Care Assessment Need (CAN) Score is a predictive analytic tool that estimates the relative probability of hospitalization and death within 90 days or one-year from the calculation date. The Office of Clinical Systems Development and Evaluation (CSDE – 10E2A) produces the weekly CAN Score Report to help identify the highest risk patients in a primary care panel or a cohort. We used the one-year score.

| **Variable** | **Definition** | **ICD Code** |
| --- | --- | --- |
| Hypertension | Two outpatient one inpatient code in past 2 years | I10, I11, I11.0, I11.9, I12.0, I12.9, I13, I13.0, I13.1, I13.10 |
| Heart Failure | Two outpatient one inpatient code in past 2 years | I09.81, I11.0, I13.0, I13.2, I50.1, I50.20, I50.21, I50.22, I50.23, I50.30, I50.31, I50.32, I50.33, I50.40, I50.41, I50.42, I50.43, I50.814, I50.82, I50.83, I50.84, I50.89, I50.9 |
| Ischemic Heart Disease | Two outpatient one inpatient code in past 2 years | I20.0, I20.8, I20.9, I21.01, I21.02, I21.09, I21.11, I21.19, I21.21, I21.29, I21.A9, I22.0, I22.1, I22.2, I22.8, I22.9, I23.0, I23.1, I23.2, I23.3, I23.4, I23.5, I23.6, I23.7, I23.8, I24.0, I24.1, I24.8, I24.9, I25.10, I25.110, I25.118, I25.119, I25.2, I25.3, I25.4, I25.5, I25.6, I25.7, I25.700, I25.708, I25.709, I25.710, I25.718, I25.719, I25.720, I25.728, I25.729, I25.730, I25.738, I25.739, I25.750, I25.758, I25.759, I25.760, I25.768, I25.769, I25.790, I25.798, I25.799, I25.810, I25.811, I25.812, I25.82, I25.83, I25.84, I25.89, I25.9, I51.2, Z98.61 |
| Diabetes | Two outpatient one inpatient code in past 2 years | E08, E08.0, E08.00, E08.01, E08.1, E08.10, E08.11, E08.2, E08.21, E08.22, E08.29, E08.3, E08.31, E08.311, E08.319, E08.32, E08.321, E08.3211, E08.3212, E08.3213, E08.3219, E08.329, E08.3291, E08.3292, E08.3293, E08.3299, E08.33, E08.331, E08.3311, E08.3312, E08.3313, E08.3319, E08.339, E08.3391, E08.3392, E08.3393, E08.3399, E08.34, E08.341, E08.3411, E08.3412, E08.3413, E08.3419, E08.349, E08.3491, E08.3492, E08.3493, E08.3499, E08.35, E08.351, E08.3511, E08.3512, E08.3513, E08.3519, E08.352, E08.3521, E08.3522, E08.3523, E08.3529, E08.353, E08.3531, E08.3532, E08.3533, E08.3539, E08.354, E08.3541, E08.3542, E08.3543, E08.3549, E08.355, E08.3551, E08.3552, E08.3553, E08.3559, E08.359, E08.3591, E08.3592, E08.3593, E08.3599, E08.36, E08.37, E08.37X1, E08.37X2, E08.37X3, E08.37X9, E08.39, E08.4, E08.40, E08.41, E08.42, E08.43, E08.44, E08.49, E08.5, E08.51, E08.52, E08.59, E08.6, E08.61, E08.610, E08.618, E08.62, E08.620, E08.621, E08.622, E08.628, E08.63, E08.630, E08.638, E08.64, E08.641, E08.649, E08.65, E08.69, E08.8, E08.9, E09, E09.0, E09.00, E09.01, E09.1, E09.10, E09.11, E09.2, E09.21, E09.22, E09.29, E09.3, E09.31, E09.311, E09.319, E09.32, E09.321, E09.3211, E09.3212, E09.3213, E09.3219, E09.329, E09.3291, E09.3292, E09.3293, E09.3299, E09.33, E09.331, E09.3311, E09.3312, E09.3313, E09.3319, E09.339, E09.3391, E09.3392, E09.3393, E09.3399, E09.34, E09.341, E09.3411, E09.3412, E09.3413, E09.3419, E09.349, E09.3491, E09.3492, E09.3493, E09.3499, E09.35, E09.351, E09.3511, E09.3512, E09.3513, E09.3519, E09.352, E09.3521, E09.3522, E09.3523, E09.3529, E09.353, E09.3531, E09.3532, E09.3533, E09.3539, E09.354, E09.3541, E09.3542, E09.3543, E09.3549, E09.355, E09.3551, E09.3552, E09.3553, E09.3559, E09.359, E09.3591, E09.3592, E09.3593, E09.3599, E09.36, E09.37, E09.37X1, E09.37X2, E09.37X3, E09.37X9, E09.39, E09.4, E09.40, E09.41, E09.42, E09.43, E09.44, E09.49, E09.5, E09.51, E09.52, E09.59, E09.6, E09.61, E09.610, E09.618, E09.62, E09.620, E09.621, E09.622, E09.628, E09.63, E09.630, E09.638, E09.64, E09.641, E09.649, E09.65, E09.69, E09.8, E09.9, E10, E10.1, E10.10, E10.11, E10.2, E10.21, E10.22, E10.29, E10.3, E10.31, E10.311, E10.319, E10.32, E10.321, E10.3211, E10.3212, E10.3213, E10.3219, E10.329, E10.3291, E10.3292, E10.3293, E10.3299, E10.33, E10.331, E10.3311, E10.3312, E10.3313, E10.3319, E10.339, E10.3391, E10.3392, E10.3393, E10.3399, E10.34, E10.341, E10.3411, E10.3412, E10.3413, E10.3419, E10.349, E10.3491, E10.3492, E10.3493, E10.3499, E10.35, E10.351, E10.3511, E10.3512, E10.3513, E10.3519, E10.352, E10.3521, E10.3522, E10.3523, E10.3529, E10.353, E10.3531, E10.3532, E10.3533, E10.3539, E10.354, E10.3541, E10.3542, E10.3543, E10.3549, E10.355, E10.3551, E10.3552, E10.3553, E10.3559, E10.359, E10.3591, E10.3592, E10.3593, E10.3599, E10.36, E10.37, E10.37X1, E10.37X2, E10.37X3, E10.37X9, E10.39, E10.4, E10.40, E10.41, E10.42, E10.43, E10.44, E10.49, E10.5, E10.51, E10.52, E10.59, E10.6, E10.61, E10.610, E10.618, E10.62, E10.620, E10.621, E10.622, E10.628, E10.63, E10.630, E10.638, E10.64, E10.641, E10.649, E10.65, E10.69, E10.8, E10.9, E11, E11.0, E11.00, E11.01, E11.1, E11.10, E11.11, E11.2, E11.21, E11.22, E11.29, E11.3, E11.31, E11.311, E11.319, E11.32, E11.321, E11.3211, E11.3212, E11.3213, E11.3219, E11.329, E11.3291, E11.3292, E11.3293, E11.3299, E11.33, E11.331, E11.3311, E11.3312, E11.3313, E11.3319, E11.339, E11.3391, E11.3392, E11.3393, E11.3399, E11.34, E11.341 E11.3411, E11.3412, E11.3413, E11.3419, E11.349, E11.3491, E11.3492, E11.3493, E11.3499, E11.35, E11.351, E11.3511, E11.3512, E11.3513, E11.3519, E11.352, E11.3521, E11.3522, E11.3523, E11.3529, E11.353, E11.3531, E11.3532, E11.3533, E11.3539, E11.354, E11.3541, E11.3542, E11.3543, E11.3549, E11.355, E11.3551, E11.3552, E11.3553, E11.3559, E11.359, E11.3591, E11.3592, E11.3593, E11.3599, E11.36, E11.37, E11.37X1, E11.37X2, E11.37X3, E11.37X9, E11.39, E11.4, E11.40, E11.41, E11.42, E11.43, E11.44, E11.49, E11.5, E11.51, E11.52, E11.59, E11.6, E11.61, E11.610, E11.618, E11.62, E11.620, E11.621, E11.622, E11.628, E11.63, E11.630, E11.638, E11.64, E11.641, E11.649, E11.65, E11.69, E11.8, E11.9, E13, E13.0, E13.00, E13.01, E13.1, E13.10, E13.11, E13.2, E13.21, E13.22, E13.29, E13.3, E13.31, E13.311, E13.319, E13.32, E13.321, E13.3211, E13.3212, E13.3213, E13.3219, E13.329, E13.3291, E13.3292, E13.3293, E13.3299, E13.33, E13.331, E13.3311, E13.3312, E13.3313, E13.3319, E13.339, E13.3391, E13.3392, E13.3393, E13.3399, E13.34, E13.341, E13.3411, E13.3412, E13.3413, E13.3419, E13.349, E13.3491, E13.3492, E13.3493, E13.3499, E13.35, E13.351, E13.3511, E13.3512, E13.3513, E13.3519, E13.352, E13.3521, E13.3522, E13.3523, E13.3529, E13.353, E13.3531, E13.3532, E13.3533, E13.3539, E13.354, E13.3541, E13.3542, E13.3543, E13.3549, E13.355, E13.3551, E13.3552, E13.3553, E13.3559, E13.359, E13.3591, E13.3592, E13.3593, E13.3599, E13.36, E13.37, E13.37X1, E13.37X2, E13.37X3, E13.37X9, E13.39, E13.4, E13.40, E13.41, E13.42, E13.43, E13.44, E13.49, E13.5, E13.51, E13.52, E13.59, E13.6, E13.61, E13.610, E13.618, E13.62, E13.620, E13.621, E13.622, E13.628, E13.63, E13.630, E13.638, E13.64, E13.641, E13.649, E13.65, E13.69, E13.8, E13.9 |
| Stroke or Transient Ischemic Attack (TIA) | Two outpatient one inpatient code in past 2 years | G45, G45.1, G45.3, G45.8, G45.9 |
| COPD or Bronchiectasis | Two outpatient one inpatient code in past 2 years | J41, J41.0, J41.1, J41.8, J42, J43, J43.0, J43.1, J43.2, J43.8, J43.9, J44, J44.0, J44.1, J44.9 |
| Cirrhosis | Two outpatient one inpatient code in past 2 years | B19.0, B19.11, B19.21, K65.2, K70.11, K70.2, K70.3, K70.30, K70.31, K70.41, K70.9, K71.11, K71.51, K71.7, K72.01, K72.11, K72.91, K74, K74.00, K74.01, K74.02, K74.1, K74.2, K74.3, K74.4, K74.5, K74.6, K74.60, K74.69, K76.6, K76.7, K76.81, I85, I85.0, I85.00, I85.01, I85.1, I85.10, I85.11, R18.8 |
| Dialysis | Any single code in prior 6 months | VA algorithm, code available upon request |
| Chronic Kidney Disease | Most recent GFR prior to booster dose | Severe CKD was defined as GFR<30 |
| Dementia | Two outpatient one inpatient code in past 2 years | F01, F01.5, F01.50, F01.51, F02, F02.8, F02.80, F02.81, F03, F03.9, F03.90, F03.91, F10.27, F10.97, F13.27, F13.97, F18.17, F18.27, F18.97, F19.17, F19.27,  F19.97, G30, G30.0, G30.1, G30.8, G30.9,  G31.0, G31.01, G31.09, G31.83 |
| Immunocompromised | 1. Receipt IV chemotherapy in the 90 days before vaccination completion 2. Receipt of immunosuppressant drugs 3. Receipt of antiretroviral drugs | See table below for complete list of drugs |
| Solid Organ Tumor | Head and Neck Cancer  Lung Cancer/respiratory tract  GI Tract Cancer  Other GI Cancer  Endocrine cancer  Genitourinary tract Cancer  Prostate cancer  CNS cancer  Bone/limb/connective tissue cancers  Other cancer | C00, C00.0, C00.1, C00.2, C00.3, C00.4, C00.5,  C00.6, C00.8, C00.9, C43.0, C4A.0, C44.00,  C44.01, C44.02, C44.09, C01, C02, C02.0, C02.1,  C02.2, C02.3, C02.4, C02.8, C02.9, C03, C03.0, C03.1, C03.9, C04, C04.0, C04.1, C04.8, C04.9, C05, C05.0, C05.1, C05.2, C05.8, C05.9, C06,  C06.0, C06.1, C06.2, C06.8, C06.80, C06.89, C06.9, C07, C08, C08.0, C08.1, C08.9, C09, C09.0, C09.1, C09.8, C09.9, C10, C10.0, C10.1, C10.2, C10.3, C10.4, C10.8, C10.9, C11, C11.0,  C11.1, C11.2, C11.3, C11.8, C11.9, C12, C13,  C13.0, C13.1, C13.2, C13.8, C13.9, C14, C14.0,  C14.2, C14.8, C30, C30.0, C30.1, C31.0, C31.1,  C31.2, C31.3, C31.8, C31.9, C32.0, C32.1, C32.2,  C32.3, C32.8, C32.9, C33, C34.00, C34.01, C34.02, C34.10, C34.11, C34.12, C34.2, C34.30,  C34.31, C34.32, C34.80, C34.81, C34.82, C34.90,  C34.91, C34.92, C37, C38.0, C38.1, C38.2, C38.3,  C38.4, C38.8, C39.0, C39.9, C31, C31.0, C31.1,  C31.2, C31.3, C31.8, C31.9, C37, C69, C69.0,  C69.00, C69.01, C69.02, C69.1, C69.10, C69.11,  C69.12, C69.2, C69.20, C69.21, C69.22, C69.3,  C69.30, C69.31, C69.32, C69.4, C69.40, C69.41,  C69.42, C69.5, C69.50, C69.51, C69.52, C69.6,  C69.60, C69.61, C69.62, C69.8, C69.80, C69.81,  C69.82, C69.9, C69.90, C69.91, C69.92, C32,  C32.0, C32.1, C32.2, C32.3, C32.8, C32.9, C33, C34, C34.0, C34.00, C34.01, C34.02, C34.1, C34.10, C34.11, C34.12, C34.2, C34.3, C34.30,  C34.31, C34.32, C34.8, C34.80, C34.81, C34.82,  C34.9, C34.90, C34.91, C34.92, C38, C38.0, C38.1, C38.2, C38.3, C38.4, C38.8, C39, C39.0, C39.9, C45, C45.0, C45.1, C45.2, C45.7, C45.9, C15, C15.3, C15.4, C15.5, C15.8, C15.9, C16, C16.0, C16.1, C16.2, C16.3, C16.4, C16.5, C16.6, C16.8, C16.9, C17, C17.0, C17.1, C17.2, C17.3,  C17.8, C17.9, C18, C18.0, C18.1, C18.2, C18.3,  C18.4, C18.5, C18.6, C18.7, C18.8, C18.9, C19, C20, C21, C21.0, C21.1, C21.2, C21.8, C22,  C22.0, C22.1, C22.2, C22.3, C22.4, C22.7, C22.8,  C22.9, C23, C24, C24.0, C24.1, C24.8, C24.9, C25, C25.0, C25.1, C25.2, C25.3, C25.4, C25.7, C25.8, C25.9, C26, C26.0, C26.1, C46.4, C26.9, C48, C48.0, C48.1, C48.2, C48.8, C73, C74,  C74.0, C74.00, C74.01, C74.02, C74.1, C74.10,  C74.11, C74.12, C74.9, C74.90, C74.91, C74.92, C7A, C7A.0, C7A.00, C7A.01, C7A.010, C7A.011,  C7A.012, C7A.019, C7A.02, C7A.020, C7A.021,  C7A.022, C7A.023, C7A.024, C7A.025, C7A.026,  C7A.029, C7A.09, C7A.090, C7A.091, C7A.092,  C7A.093, C7A.094, C7A.095, C7A.096, C7A.098,  C7A.1, C7A.8, C7B, C7B.0, C7B.00, C7B.01,  C7B.02, C7B.03, C7B.04, C7B.09, C7B.1, C7B.8, C75, C75.0, C75.1, C75.2, C75.3, C75.4, C75.5,  C75.8, C75.9, C62, C62.0, C62.00, C62.01, C62.02, C62.1, C62.10, C62.11, C62.12, C62.9,  C62.90, C62.91, C62.92, C63, C63.0, C63.00,  C63.01, C63.02, C63.1, C63.10, C63.11, C63.12,  C63.2, C63.7, C63.8, C63.9, C64, C64.1, C64.2,  C64.9, C65, C65.1, C65.2, C65.9, C67, C67.0,  C67.1, C67.2, C67.3, C67.4, C67.5, C67.6, C67.7,  C67.8, C67.9, C66, C66.1, C66.2, C66.9, C68,  C68.0, C68.1, C68.8, C68.9, C61, C70, C70.0,  C70.1, C70.9, C71, C71.0, C71.1, C71.2, C71.3,  C71.4, C71.5, C71.6, C71.7, C71.8, C71.9, C72,  C72.0, C72.1, C72.2, C72.20, C72.21, C72.22,  C72.3, C72.30, C72.31, C72.32, C72.4, C72.40,  C72.41, C72.42, C72.5, C72.50, C72.59, C72.9,  C40, C40.0, C40.00, C40.01, C40.02, C40.1, C40.10, C40.11, C40.12, C40.2, C40.20, C40.21,  C40.22, C40.3, C40.30, C40.31, C40.32, C40.8,  C40.80, C40.81, C40.82, C40.9, C40.90, C40.91,  C40.92, C41, C41.0, C41.1, C41.2, C41.3, C41.4, C41.9, C46, C46.0, C46.1, C46.2, C46.3, C46.4, C46.5, C46.50, C46.51, C46.52, C46.7, C46.9, C47, C47.0, C47.1, C47.10, C47.11, C47.12, C47.2, C47.20, C47.21, C47.22, C47.3, C47.4, C47.5, C47.6, C47.8, C47.9, C49, C49.0, C49.1, C49.10, C49.11, C49.12, C49.2, C49.20, C49.21, C49.22, C49.3, C49.4, C49.5, C49.6, C49.8, C49.9, C49.A, C49.A0, C49.A1, C49.A2, C49.A3, C49.A4, C49.A5, C49.A9, C76, C76.0, C76.1, C76.2, C76.3, C76.4, C76.40, C76.41, C76.42, C76.5, C76.50, C76.51, C76.52,  C76.8, C79, C79.0, C79.00, C79.01, C79.02,  C79.1, C79.10, C79.11, C79.19, C79.2, C79.3,  C79.31, C79.32, C79.4, C79.40, C79.49, C79.5,  C79.51, C79.52, C79.6, C79.60, C79.61, C79.62,  C79.7, C79.70, C79.71, C79.72, C79.8, C79.81,  C79.82, C79.89, C79.9, C78, C78.0, C78.00,  C78.01, C78.02, C78.1, C78.2, C78.3, C78.30,  C78.39, C78.4, C78.5, C78.6, C78.7, C78.8, C78.80, C78.89, C77, C77.0, C77.1, C77.2, C77.3, C77.4, C77.5, C77.8, C77.9, C80, C80.0, C80.1,  C80.2 |
| Hematologic malignancy | Hodgkin lymphoma  Follicular lymphoma  Non follicular lymphoma  TNK cell lymphoma  Other non-Hodgkin’s lymphoma  Other specified TNK lymphoma  Immunoproliferative and b cell lymphoma  Multiple myeloma and plasma cell  Lymphoid leukemia  Myeloid leukemia  Monocytic leukemia  Leukemia & Specified Cell Type  Leukemia of unspecified cell  Unspecified malignant neoplasm | C81, C81.0, C81.00, C81.01, C81.02, C81.03,  C81.04, C81.05, C81.06, C81.07, C81.08, C81.09,  C81.1, C81.10, C81.11, C81.12, C81.13, C81.14,  C81.15, C81.16,  C81.17, C81.18, C81.19, C81.2, C81.20, C81.21, C81.22, C81.23, C81.24, C81.25,  C81.26, C81.27, C81.28, C81.29, C81.3, C81.30, C81.31, C81.32, C81.33, C81.34, C81.35, C81.36,  C81.37, C81.38, C81.39, C81.4, C81.40, C81.41, C81.42, C81.43, C81.44, C81.45, C81.46, C81.47, C81.48, C81.49, C81.7, C81.70, C81.71, C81.72, C81.73, C81.74, C81.75, C81.76, C81.77, C81.78, C81.79, C81.9, C81.90, C81.91, C81.92, C81.93, C81.94, C81.95, C81.96, C81.97, C81.98, C81.99, C82, C82.0, C82.00, C82.01, C82.02, C82.03, C82.04, C82.05, C82.06, C82.07, C82.08, C82.09, C82.1, C82.10, C82.11, C82.12, C82.13, C82.14, C82.15, C82.16, C82.17, C82.18, C82.19, C82.2, C82.20, C82.21, C82.22, C82.23, C82.24, C82.25, C82.26, C82.27, C82.28, C82.29, C82.3, C82.30, C82.31, C82.32, C82.33, C82.34, C82.35, C82.36, C82.37, C82.38, C82.39, C82.4, C82.40, C82.41, C82.42, C82.43, C82.44, C82.45, C82.46, C82.47, C82.48, C82.49, C82.5, C82.50, C82.51, C82.52, C82.53, C82.54, C82.55, C82.56, C82.57, C82.58, C82.59, C82.6, C82.60, C82.61, C82.62, C82.63, C82.64, C82.65, C82.66, C82.67, C82.68, C82.69, C82.8, C82.80, C82.81, C82.82, C82.83, C82.84,  C82.85, C82.86, C82.87, C82.88, C82.89, C82.9,  C82.90, C82.91, C82.92, C82.93, C82.94, C82.95,  C82.96, C82.97, C82.98, C82.99, C83, C83.0,  C83.00, C83.01, C83.02, C83.03, C83.04, C83.05,  C83.06, C83.07, C83.08, C83.09, C83.1, C83.10,  C83.11, C83.12, C83.13, C83.14, C83.15, C83.16,  C83.17, C83.18, C83.19, C83.3, C83.30, C83.31,  C83.32, C83.33, C83.34, C83.35, C83.36, C83.37,  C83.38, C83.39, C83.5, C83.50, C83.51, C83.52,  C83.53, C83.54, C83.55, C83.56, C83.57, C83.58,  C83.59, C83.7, C83.70, C83.71, C83.72, C83.73,  C83.74, C83.75, C83.76, C83.77, C83.78, C83.79,  C83.8, C83.80, C83.81, C83.82, C83.83, C83.84,  C83.85, C83.86, C83.87, C83.88, C83.89, C83.9,  C83.90, C83.91, C83.92, C83.93, C83.94, C83.95,  C83.96, C83.97, C83.98, C83.99, C84, C84.0, C84.00, C84.01, C84.02, C84.03, C84.04, C84.05,  C84.06, C84.07, C84.08, C84.09, C84.1, C84.10,  C84.11, C84.12, C84.13, C84.14, C84.15, C84.16,  C84.17, C84.18, C84.19, C84.4, C84.40, C84.41,  C84.42, C84.43, C84.44, C84.45, C84.46, C84.47,  C84.48, C84.49, C84.6, C84.60, C84.61, C84.62,  C84.63, C84.64, C84.65, C84.66, C84.67, C84.68,  C84.69, C84.7, C84.70, C84.71, C84.72, C84.73,  C84.74, C84.75, C84.76, C84.77, C84.78, C84.79,  C84.A, C84.A0, C84.A1, C84.A2, C84.A3, C84.A4,  C84.A5, C84.A6, C84.A7, C84.A8, C84.A9, C84.Z,  C84.Z0, C84.Z1, C84.Z2, C84.Z3, C84.Z4, C84.Z5,  C84.Z6, C84.Z7, C84.Z8, C84.Z9, C84.9, C84.90,  C84.91, C84.92, C84.93, C84.94, C84.95, C84.96,  C84.97, C84.98, C84.99, C85, C85.1, C85.10,  C85.11, C85.12, C85.13, C85.14, C85.15, C85.16,  C85.17, C85.18, C85.19, C85.2, C85.20, C85.21,  C85.22, C85.23, C85.24, C85.25, C85.26, C85.27,  C85.28, C85.29, C85.8, C85.80, C85.81, C85.82,  C85.83, C85.84, C85.85, C85.86, C85.87, C85.88,  C85.89, C85.9, C85.90, C85.91, C85.92, C85.93,  C85.94, C85.95, C85.96, C85.97, C85.98, C85.99, C86, C86.0, C86.1, C86.2, C86.3, C86.4, C86.5,  C86.6, C88, C88.0, C88.2, C88.3, C88.4, C88.8,  C88.9, C90, C90.0, C90.00, C90.01, C90.02,  C90.1, C90.10, C90.11, C90.12, C90.2, C90.20,  C90.21, C90.22, C90.3, C90.30, C90.31, C90.32, C91, C91.0, C91.00, C91.01, C91.02, C91.1,  C91.10, C91.11, C91.12, C91.3, C91.30, C91.31,  C91.32, C91.4, C91.40, C91.41, C91.42, C91.5,  C91.50, C91.51, C91.52, C91.6, C91.60, C91.61,  C91.62, C91.A, C91.A0, C91.A1, C91.A2, C91.Z,  C91.Z0, C91.Z1, C91.Z2, C91.9, C91.90, C91.91,  C91.92, C92 C92.0, C92.00, C92.01, C92.02,  C92.1, C92.10, C92.11, C92.12, C92.2, C92.20,  C92.21, C92.22, C92.3, C92.30, C92.31, C92.32,  C92.4, C92.40, C92.41, C92.42, C92.5, C92.50,  C92.51, C92.52, C92.6, C92.60, C92.61, C92.62,  C92.A, C92.A0, C92.A1, C92.A2, C92.Z, C92.Z0,  C92.Z1, C92.Z2, C92.9, C92.90, C92.91, C92.92, C93, C93.0, C93.00, C93.01, C93.02, C93.1,  C93.10, C93.11, C93.12, C93.3, C93.30, C93.31,  C93.32, C93.Z, C93.Z0, C93.Z1, C93.Z2, C93.9,  C93.90, C93.91, C93.92, C94, C94.0, C94.00,  C94.01, C94.02, C94.2, C94.20, C94.21, C94.22,  C94.3, C94.30, C94.31, C94.32, C94.4, C94.40,  C94.41, C94.42, C94.6, C94.8, C94.80, C94.81,  C94.82, C95, C95.0, C95.00, C95.01, C95.02,  C95.1, C95.10, C95.11, C95.12, C95.9, C95.90,  C95.91, C95.92, C96, C96.0, C96.2, C96.20,  C96.21, C96.22, C96.29, C96.4, C96.5, C96.6,  C96.A |
| Current smoker | Corporate Data Warehouse health factors file and existing algorithm | ICD-10 codes, at least one code in the past year  F17.200, F17.210, F17.290, Z72.0  Tobacco use CPT code, at least one code in the past year:  99406, 99407, S9075,  S9453, G0436, G0437  Clinic stop code, at least one code in the past year:  707, 708  OR  Most recent health factor within past year indicates current smoker. Table of smoking-related health factors adapted from table available at: https://medicine.yale.edu/intmed/vacs/ |
| Alcohol Use Disorder | Any single ICD code in past year | F10.10, F10.120, F10.121, F10.129, F10.14,  F10.150, F10.151, F10.159, F10.180, F10.181, F10.182, F10.188, F10.19, F10.20, F10.220, F10.221, F10.229, F10.230, F10.231, F10.232, F10.239, F10.24, F10.250, F10.251, F10.259, F10.26, F10.27, F10.280, F10.281, F10.282, F10.288, F10.29, F10.920, F10.921, F10.929, F10.94, F10.950, F10.951, F10.959, F10.96, F10.97, F10.980, F10.981, F10.982, F10.988, F10.99, G31.2, G62.1, I42.6, K29.20, K29.21, K70.0, K70.10, K70.11, K70.2, K70.30, K70.31, K70.40, K70.41, K70.9, O35.4XX0, O35.4XX1, O35.4XX2, O35.4XX3, O35.4XX4, O35.4XX5, O35.4XX9, O99.310, O99.311, O99.312, O99.313, O99.314, O99.315, Z71.4, Z71.41 |
| Drug Use Disorder | Any single ICD code in past year | F11.10, F11.120, F11.121, F11.122, F11.129, F11.13, F11.14, F11.150, F11.151, F11.159, F11.181, F11.182, F11.188, F11.19, F11.20, F11.21, F11.220, F11.221, F11.222, F11.229, F11.23, F11.24, F11.250, F11.251, F11.259, F11.281, F11.282, F11.288, F11.29, F11.90, F11.920, F11.921, F11.922, F11.929, F11.93, F11.94, F11.950, F11.951, F11.959, F11.981, F11.982, F11.988, F11.99, F12.10, F12.120, F12.121, F12.122, F12.129, F12.150, F12.151, F12.159, F12.180, F12.188, F12.19, F12.20, F12.21, F12.220, F12.221, F12.222, F12.229, F12.23, F12.250, F12.251, F12.259, F12.280, F12.288, F12.29, F12.90, F12.920, F12.921, F12.922, F12.929, F12.93, F12.950, F12.951, F12.959, F12.980, F12.988, F12.99, F13.10, F13.120, F13.121, F13.129, F13.14, F13.150, F13.151, F13.159, F13.180, F13.181, F13.182, F13.188, F13.19, F13.20, F13.21, F13.220, F13.221, F13.229, F13.230, F13.231, F13.232, F13.239, F13.24, F13.250, F13.251, F13.259, F13.26, F13.27, F13.280, F13.281, F13.282, F13.288, F13.29, F13.90, F13.920, F13.921, F13.929, F13.930, F13.931, F13.932, F13.939, F13.94, F13.950, F13.951, F13.959, F13.96, F13.97, F13.980, F13.981, F13.982, F13.988, F13.99, F14.10, F14.120, F14.121, F14.122, F14.129, F14.13, F14.14, F14.150, F14.151, F14.159, F14.180, F14.181, F14.182, F14.188, F14.19, F14.20, F14.21, F14.220, F14.221, F14.222, F14.229, F14.23, F14.24, F14.250, F14.251, F14.259, F14.280, F14.281, F14.282, F14.288, F14.29, F14.90, F14.920, F14.921, F14.922, F14.929, F14.93, F14.94, F14.950, F14.951, F14.959, F14.980, F14.981, F14.982, F14.988, F14.99, F15.10, F15.120, F15.121, F15.122, F15.129, F15.13, F15.14, F15.150, F15.151, F15.182, F15.188, F15.19, F15.20, F15.21, F15.220, F15.221, F15.222, F15.229, F15.23, F15.24, F15.250, F15.251, F15.259, F15.280, F15.281, F15.282, F15.288, F15.29, F15.90, F15.920, F15.921, F15.922, F15.929, F15.93, F15.94, F15.950, F15.951, F15.959, F15.980, F15.981, F15.982, F15.988, F15.99, F16.10, F16.120, F16.121, F16.122, F16.129, F16.14, F16.150, F16.151, F16.159, F16.180, F16.183, F16.188, F16.19, F16.20, F16.21, F16.220, F16.221, F16.229, F16.24, F16.250, F16.251, F16.259, F16.280, F16.283, F16.288, F16.29, F16.90, F16.920, F16.921, F16.929, F16.94, F16.950, F16.951, F16.959, F16.980, F16.983, F16.988, F16.99, F18.10, F18.120, F18.121, F18.129, F18.14, F18.150, F18.151, F18.159, F18.17, F18.180, F18.188, F18.19, F18.20, F18.21, F18.220, F18.221, F18.229, F18.24, F18.250, F18.251, F18.259, F18.27, F18.280, F18.288, F18.29, F18.90, F18.920, F18.921, F18.929, F18.94, F18.950, F18.951, F18.959, F18.97, F18.980, F18.988, F18.99, F19.10, F19.120, F19.121, F19.122, F19.129, F19.130, F19.131, F19.132, F19.139, F19.14, F19.150, F19.151, F19.159, F19.16, F19.17, F19.180, F19.181, F19.182, F19.188, F19.19, F19.20, F19.21, F19.220, F19.221, F19.222, F19.229, F19.230, F19.231, F19.232, F19.239, F19.24, F19.250, F19.251, F19.259, F19.26, F19.27, F19.280, F19.281, F19.282, F19.288, F19.29, F19.90, F19.920, F19.921, F19.922, F19.929, F19.930, F19.931, F19.932, F19.939, F19.94, F19.950, F19.951, F19.959, F19.96, F19.97, F19.980, F19.981, F19.982, F19.988, F19.99 |
| Marginally Housed | ICD codes or clinic stop codes related to marginal housing | ICD-10 codes, at least one code in past two years Z59.0 Homelessness Z59.1 Inadequate housing Z59.8 Other problems related to housing and economic circumstances Z59.9 Problem related to housing and economic circumstances, unspecified OR  Clinic Stop codes, at least one code in past two years: 507, 522, 528, 529, 530 |
| Body Mass Index | Used more recent measurement before index date available in the Corporate Data Warehouse |  |
| VA Priority score | VA Priority Groups | <https://www.va.gov/health-care/eligibility/priority-groups/> |

**STable 3. Characteristics of propensity-score matched cohort. Propensity score includes time since last immunological event. All standardized differences were all < 0.1 after matching.**

|  | **Total Cohort** | **Received Variant-target Booster** | **Did Not Receive Variant-target Booster Matched Pair** | **Standard Deviation** |
| --- | --- | --- | --- | --- |
| **N** | 1,576,626 | 788,313 | 788,313 |  |
| **Gender (N, %)** |  |  |  |  |
| Male | 1,458,718 (92.5) | 730,054 (92.6) | 728,664 (92.4) | -0.0018 |
| Female | 117,908 (7.5) | 58,259 (7.4) | 59,649 (7.6) | 0.0018 |
| **Age (mean, SD)** |  | 71.4 (11.1) | 71.4 (11.1) | 0.0000 |
| **Age (N, %)** |  |  |  |  |
| 18-64 | 342,160 (21.7) | 171,080 (21.7) | 171,080 (21.7) | 0.0000 |
| 65-74 | 561,310 (35.6) | 280,655 (35.6) | 280,655 (35.6) | 0.0000 |
| 75-84 | 526,912 (33.4) | 263,456 (33.4) | 263,456 (33.4) | 0.0000 |
| 85 or older | 146,244 (9.3) | 73,122 (9.3) | 73,122 (9.3) | 0.0000 |
| **Race^a^ (N, %)** |  |  |  |  |
| American Indian or Alaska Native | 10,139 (0.6) | 5,023 (0.6) | 5,116 (0.6) | 0.0001 |
| Asian | 22,449 (1.4) | 11,002 (1.4) | 11,447 (1.5) | 0.0006 |
| Black or African American | 295,699 (18.8) | 147,982 (18.8) | 147,717 (18.7) | -0.0003 |
| Native Hawaiian or other Pacific Islander | 12,757 (0.8) | 6,259 (0.8) | 6,498 (0.8) | 0.0003 |
| White | 1,118,890 (71.0) | 56,0662 (71.1) | 55,8228 (70.8) | -0.0031 |
| More than one race | 11,452 (0.7) | 5,622 (0.7) | 5,830 (0.7) | 0.0003 |
| Missing | 105,240 (6.7) | 51,763 (6.6) | 53,477 (6.8) | 0.0022 |
| **Ethnicity^a^ (N, %) -- Hispanic or Latino** | 93,906 (6.0) | 45,502 (5.8) | 48,404 (6.1) | 0.0037 |
| **Marital Status (N, %) -- Currently married** | 962,968 (61.1) | 484,062 (61.4) | 478,906 (60.8) | -0.0065 |
| **Urban/rural^b^ (N, %)** |  |  |  |  |
| Highly rural or unknown | 66,385 (4.2) | 32,717 (4.2) | 33,668 (4.3) | 0.0012 |
| Rural | 414,754 (26.3) | 206,650 (26.2) | 208,104 (26.4) | 0.0018 |
| Urban | 1,095,487 (69.5) | 548,946 (69.6) | 546,541 (69.3) | -0.0031 |
| **BMI (N, %)** |  |  |  |  |
| <18.5 | 10,388 (0.7) | 5,053 (0.6) | 5,335 (0.7) | 0.0004 |
| 18.5-24.9 | 260,298 (16.5) | 127,589 (16.2) | 132,709 (16.8) | 0.0065 |
| 25-29.9 | 547,237 (34.7) | 274,554 (34.8) | 272,683 (34.6) | -0.0024 |
| >=30 | 643,746 (40.8) | 324,349 (41.1) | 319,397 (40.5) | -0.0063 |
| Missing | 114,957 (7.3) | 56,768 (7.2) | 58,189 (7.4) | 0.0018 |
| **Comorbidities associated with severe COVID-19 illness (N, %)** |  |  |  |  |
| Hypertension | 1,133,663 (71.9) | 568,789 (72.2) | 564,874 (71.7) | -0.0050 |
| Diabetes | 587,477 (37.3) | 295,014 (37.4) | 292,463 (37.1) | -0.0032 |
| **CKD^d^** |  |  |  |  |
| CKD | 326,063 (20.7) | 160,718 (20.4) | 165,345 (21.0) | 0.0059 |
| No CKD | 1,216,969 (77.2) | 611,238 (77.5) | 605,731 (76.8) | -0.0070 |
| Severe CKD | 33,594 (2.1) | 16,357 (2.1) | 17,237 (2.2) | 0.0011 |
| Ischemic Heart Disease | 377,508 (23.9) | 187,382 (23.8) | 190,126 (24.1) | 0.0035 |
| COPD Bronchiectasis | 234,510 (14.9) | 116,122 (14.7) | 118,388 (15.0) | 0.0029 |
| CHF | 144,398 (9.2) | 70,737 (9.0) | 73,661 (9.3) | 0.0037 |
| Immunocompromised^e^ | 100,635 (6.4) | 49,838 (6.3) | 50,797 (6.4) | 0.0012 |
| Stroke TIA | 68,088 (4.3) | 33,394 (4.2) | 34,694 (4.4) | 0.0016 |
| Dementia | 42,589 (2.7) | 20,299 (2.6) | 22,290 (2.8) | 0.0025 |
| Cirrhosis | 31,184 (2.0) | 15,645 (2.0) | 15,539 (2.0) | -0.0001 |
| Cancer lymphoma leukemia^f^ | 27,206 (1.7) | 13,394 (1.7) | 13,812 (1.8) | 0.0005 |
| Dialysis | 11,737 (0.7) | 5,819 (0.7) | 5,918 (0.8) | 0.0001 |
| Cancer other^f^ | 17,989 (1.1) | 9,204 (1.2) | 8,785 (1.1) | -0.0005 |
| Spinal cord injury | 9,762 (0.6) | 4,910 (0.6) | 4,852 (0.6) | -0.0001 |
| **Social and behavioral risk factors (N, %)** |  |  |  |  |
| Current smoker | 275,182 (17.5) | 135,472 (17.2) | 139,710 (17.7) | 0.0054 |
| Alcohol abuse^i^ | 113,897 (7.2) | 56,343 (7.1) | 57,554 (7.3) | 0.0015 |
| Substance use^j^ | 81,374 (5.2) | 40,348 (5.1) | 41,026 (5.2) | 0.0009 |
| Housing problems^k^ | 57,969 (3.7) | 28,674 (3.6) | 29,295 (3.7) | 0.0008 |
| **VA priority^l^ (N, %)** |  |  |  |  |
| 1 | 652,744 (41.4) | 328,543 (41.7) | 324,201 (41.1) | -0.0055 |
| 2 | 110,617 (7.0) | 55,010 (7.0) | 55,607 (7.1) | 0.0008 |
| 3 | 219,714 (13.9) | 110,822 (14.1) | 108,892 (13.8) | -0.0024 |
| 4 | 15,244 (1.0) | 7,293 (0.9) | 7,951 (1.0) | 0.0008 |
| 5 | 228,830 (14.5) | 112,904 (14.3) | 115,926 (14.7) | 0.0038 |
| 6 | 71,110 (4.5) | 36,341 (4.6) | 34,769 (4.4) | -0.0020 |
| 7 | 67,451 (4.3) | 33,435 (4.2) | 34,016 (4.3) | 0.0007 |
| 8 | 210,027 (13.3) | 103,509 (13.1) | 106,518 (13.5) | 0.0038 |
| Missing | 889 (0.1) | 456 (0.1) | 433 (0.1) | 0.0000 |
| **CAN score^m^ (N, %)** |  |  |  |  |
| 0-24.9 | 129,033 (8.2) | 61,439 (7.8) | 67,594 (8.6) | 0.0078 |
| 25-49.9 | 349,863 (22.2) | 177,746 (22.5) | 172,117 (21.8) | -0.0071 |
| 50-74.9 | 542,478 (34.4) | 277,209 (35.2) | 265,269 (33.7) | -0.0151 |
| 75 - 100 | 534,682 (33.9) | 261,306 (33.1) | 273,376 (34.7) | 0.0153 |
| Missing | 20,570 (1.3) | 10,613 (1.3) | 9,957 (1.3) | -0.0008 |
| Home-based primary care | 24,846 (1.6) | 11,799 (1.5) | 13,047 (1.7) | 0.0016 |
| **Reinfection with COVID-19^h^ (N, %)** |  |  |  |  |
| Pre-Omicron | 111,393 (7.1) | 56,457 (7.2) | 54,936 (7.0) | -0.0019 |
| Omicron | 67,031 (4.3) | 25,491 (3.2) | 41,540 (5.3) | 0.0204 |
| No prior infection | 1,398,202 (88.7) | 706,365 (89.6) | 691,837 (87.8) | -0.0184 |
| **Time period of prior booster (N, %)** |  |  |  |  |
| September 1 - December 15, 2022 | -- | 672,277 (85.3) | -- |  |
| After December 15, 2022 | -- | 116,036 (14.7) | -- |  |
| ^a^Race/ethnicity was assessed using self-identified data found in Veteran Health Records.  ^b^Urban/rural was assessed using defined based on the Rural Urban Commuting Area (RUCA) categories developed by the Department of Agriculture and Health and Human Services’ Health Resource and Services Administration.  ^c^CKD defined as having a glomerular filtration rate between 30 and 60.  ^d^Severe CKD defined as having a glomerular filtration rate <30.  ^e^Immunocompromised definition based on medications and history of cancer (see supplement for list of meds).  ^f^Cancer definition based on diagnosis codes. 2 outpatient or 1 inpatient diagnosis code in the VA (see supplement).  ^g^Alcohol use defined as1 outpatient or 1 inpatient code within 2 years of index.  ^h^Including cannabis, opioids, inhalants.  ^i^Housing problems defined as homelessness, inadequate housing, other problems related to housing and economic circumstances.  ^j^VA-defined based on factors, iiincluding military service history, disability rating and, income level to identify Veterans to determine enrolment priority; score ranges from 1 to 8 with 1 being the highest priority. This is a surrogate for socioeconomic status.  ^k^The Care Assessment Need (CAN) Score is a predictive analytic tool that estimates the relative probability of hospitalization and death within 90 days or one-year from the calculation date. The Office of Clinical Systems Development and Evaluation (CSDE – 10E2A) produces the weekly CAN Score Report to help identify the highest risk patients in a primary care panel or a cohort. We used the one-year score.  ^l^Last documented infection occurred 91 or more days prior to boosted shot.  Abbreviations: BMI: body mass index; CHF: chronic heart failure; CKD: chronic kidney disease; COPD: chronic obstructive pulmonary disease; TIA: transient ischemic attack | | | | |
| Note: n = 298 patients who received bivalent vaccine were matched on their date of receiving bivalent vaccine because they experienced the outcome (pna) first. | | | | |

**SFigure 2. Kaplan-Meier survival curve of hospitalization 0 to 7 days after vaccination to evaluate the negative outcome control. Survival probabilities were similar between groups.**


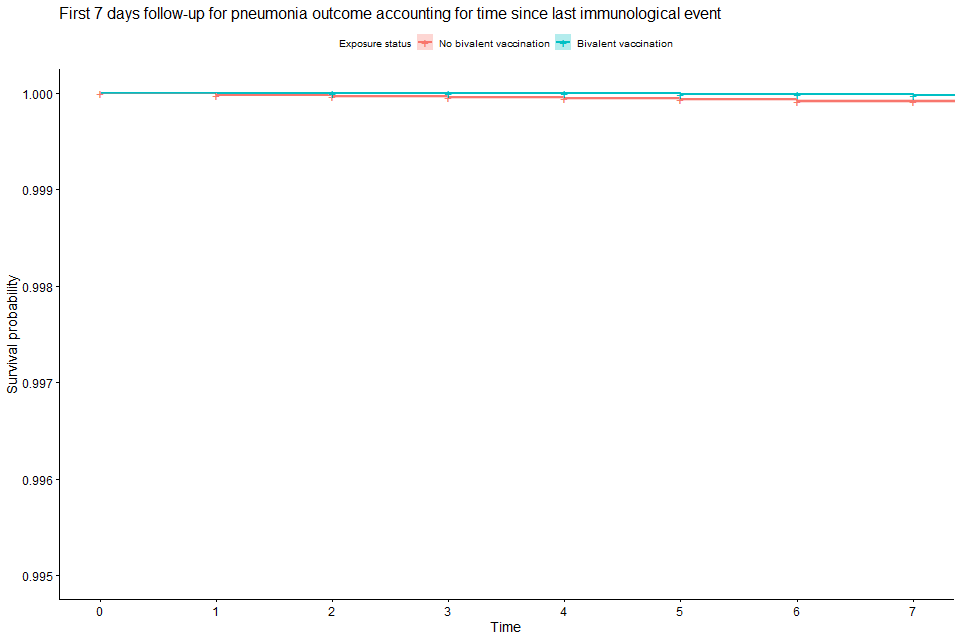


**SFigure 3. Flow Diagram**


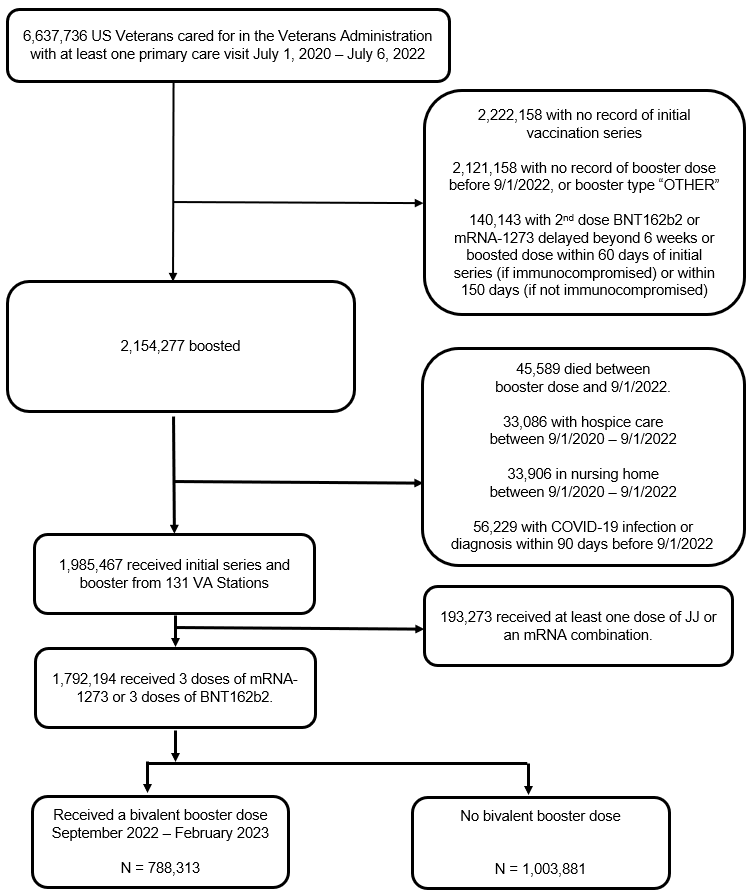


**STable 4ab. Relative vaccine effectiveness for variant-targeted mRNA booster effectiveness against hospitalization due to COVID-19 pneumonia and in-hospital severe illness, stratified analyses by receipt of the booster dose before and during the XBB Omicron variant predominant era. Patients vaccinated before January 1, 2023 were part of pre-XBB era, and those vaccinated after January 1, 2023 were part of XBB era. These relative vaccine effectiveness estimates represent estimates matched on propensity score, including time since last booster, from two cohorts (pre-XBB, XBB era) and followed until the end of the era under observation.**

| **STable 4a: Overall cohort (vaccine-derived immunity)** | | | | |
| --- | --- | --- | --- | --- |
| **Hospitalization due to COVID-19 Pneumonia** | Relative VE | 95% CI | *p*-value |  |
| Vaccinated before 1/1/23 | 30% | (25%, 34%) | <0.01 |  |
| Vaccinated on or after 1/1/23 | 32% | (14%, 46%) | <0.01 |  |
| **In-hospital Severe Illness** |  |  |  |  |
| Vaccinated before 1/1/23 | 32% | (22%, 40%) | <0.01 |  |
| Vaccinated on or after 1/1/23 | 17% | (-22%, 43%) | 0.35 |  |
| Abbreviations: CI: Confidence Interval; VE: vaccine effectiveness | | | |  |
|  |  |  |  |  |
|  |  |  |  |  |
| **STable 4b: Cohort limited to patients with prior infection in Pre-Omicron or Omicron periods (population with hybrid immunity)** | | | |  |
| **Hospitalization due to COVID-19 Pneumonia** | Relative VE | 95% CI | *p*-value |  |
| Vaccinated before 1/1/23 | 38% | (27%, 48%) | <0.01 |  |
| Vaccinated on or after 1/1/23 | 39% | (-6%, 65%) | 0.08 |  |
| **In-hospital Severe Illness** |  |  |  |  |
| Vaccinated before 1/1/23 | 52% | (31%, 67%) | <0.01 |  |
| Vaccinated on or after 1/1/23 | 33% | (-66%, 73%) | 0.38 |  |
| Abbreviations: CI: Confidence Interval; VE: vaccine effectiveness | | | |  |

**STable 5. Immunocompromised status defined based on receipt of three classes of drugs**

(1) Receipt IV chemotherapy in the 90 days before vaccination completion

(2) Receipt of immunosuppressive drugs

(3) Receipt of antiretroviral drugs

| Chemotherapy | Bendamustine, Busulfan, Carboplatin, Carmustine, Chlorambucil, Cisplatin, Cyclophosphamide, Dacarbazine, Estramustine, Ifosfamide, Lomustine, Mechlorethamine, Melphalan, Oxaliplatin, Pipobroman, Procarbazine, Streptozocin, Temozolomide, Thiotepa, Uracil Mustard, Azathioprine, Capecitabine, Cladribine, Clofarabine, Cytarabine, Floxuridine, Fludarabine, Fluorouracil, Gemcitabine, Hydroxyurea, Mercaptopurine, Methotrexate, Pentostatin, Pralatrexate, Thioguanine, Vidarabine, Daunorubicin, Doxorubicin, Epirubicin, Idarubicin, Pixantrone, Valrubicin, Vincristine, Vinblastine, Vinorelbine, Etoposide, Teniposide, Irinotecan, Mitoxantrone, Topotecan, Paclitaxel, Docetaxel |
| --- | --- |
| Immunosuppressive drugs | Abatacept, Adalimumab, Anakinra, Belimumab, Canakinumab, Certolizumab, Denosumab, Eculizumab, Etanercept, Golimumab, Infliximab, Natalizumab, Rituximab, Secukinumab, Siltuximab, Tocilizumab, Sarilumab, Ustekinumab, Vedolizumab, Tofacitinib, Baricitinib, Upadacitinib, Apremilast, Mercaptopurine, Azathioprine, Cyclophosphamide, Cyclosporine, Hydroxychloroquine, Chloroquine, Leflunomide, Methotrexate, Mycophenolate mofetil, Mycophenolic acid, Sulfasalazine, Tacrolimus, Chloroquine, Hydroxychloroquine, Prednisone, Dexamethasone, Hydrocortisone, Methylprednisolone, Cortisone, Prednisolone, Triamcinolone, Betamethasone |
| Antiretroviral drugs | Abacavir (ABC), Didanosine (ddI), Emtricitabine (FTC), Lamivudine (3TC), Stavudine (d4T), Tenofovir (TDF), Zalcitabine (ddC), Zidovudine (ZDV, AZT), Delavirdine (DLV), Efavirenz (EFV), Etravirine (ETR), Nevirapine (NVP), Rilpivirine (RPV), Amprenavir (APV), Atazanavir (ATV), Atazanavir-cobicistat (ATV/COBI), Darunavir (DRV), Darunavir-cobicistat (DRV/COBI), Fosamprenavir (FPV), Indinavir (IDV), Lopinavir/ritonavir (LPV/r), Nelfinavir (NFV), Ritonavir (RTV), Saquinavir (SQV), Tipranavir (TPV), Enfuvirtide (T-20), Dolutegravir (DTG), Elvitegravir (EVG), Raltegravir (RAL), Maraviroc (MVC) |
